# Gut microbiota regulates growth retardation in pigs through their metabolites of taurine and butyric acids

**DOI:** 10.3389/fmicb.2026.1811659

**Published:** 2026-04-17

**Authors:** Tao Shen, Yunyan Zhou, Jun Gao, Xinwei Xiong, Congying Chen

**Affiliations:** National Key Laboratory of Swine Genetic Improvement and Germplasm Innovation, Jiangxi Agricultural University, Nanchang, China

**Keywords:** butyric acid, growth retardation, gut microbiome, metabolome, pigs, taurine

## Abstract

Growth retardation of piglets has always been observed in current pig production system. Here we defined these pigs as stunted pigs. Stunted pigs show normal feed intake, but exhibit extremely slow growth speed. This brings a big economic loss to pig industry. Many factors can lead to growth retardation, including gut microbiota which has been reported to play important roles in growth retardation of children. However, whether and which gut microbial taxa are associated with growth retardation of piglets are largely unknown. Here we used 16S rRNA gene and shotgun metagenomic sequencing to identify bacterial taxa associated with growth retardation in 126 pigs including stunted pigs and their pairwise littermates showing normal growth. We identified several *Clostridium* spp. significantly enriched in the gut of normal growing pigs, including *Clostridium symbiosum* which was the key biomarker distinguishing stunted pigs and normal growing pigs, while several *Bacteroides* spp. had higher abundances in stunted pigs. *Clostridium* spp. was significantly associated with the shifts of functional capacities of the gut microbiome between normal and stunted pigs, e.g., biosynthesis of unsaturated fatty acids. Untargeted serum metabolome analysis found that normal growing pigs had higher concentration of taurine in serum. Increased concentration of serum taurine was associated with increased abundance of *Clostridium symbiosum*. Furthermore, all metabolites having higher abundances in normal growing pigs were enriched in the pathway of taurine and hypotaurine metabolism. Short-chain fatty acids (SCFAs) analysis identified butyric acid having higher concentration in feces of normal growing pigs in both discovery and validation cohorts, and the changes in the abundances of *Clostridium symbiosum* was correlated with the shifts of the concentrations of fecal SCFAs. These results suggested that *Clostridium* spp., especially *Clostridium symbiosum* improved pig growth by increasing the concentrations of serum taurine and fecal butyric acid, and was an important biomarker associated with pig growth. This study provided important insights into the effect of the gut microbiome on pig growth retardation.

## Introduction

Stunted pigs have always represented those pigs which showing growth retardation or extremely low daily body weight gain, but exhibiting normal mental states, appetite and daily feed intake. Compared with their pairwise littermates with normal growth speed, stunted pigs have distinctly small body size and weight. The incidence of stunted pigs in farms is about 1–3%. It brings a big economic loss to pig industry. Many factors can lead to the incidence of stunted pigs, including lactation and nursing, diets provided to piglets, and diseases. If sows cannot produce enough milk for piglets or show poor lactation ability, the nutrition cannot fit the requirements of suckling piglets, which further results in the growth retardation (Prendergast and Humphrey, 2014; George et al., 2018). Diseases are another important factor leading to growth retardation. For example, chronic gastroenterias impedes piglet growth (Naylor et al., 2015). All these factors suggested that the dysbacteriosis of the gut microbiota should be involved in the generation of stunted pigs.

In recent years, more and more studies have reported the correlations between the composition of gut microbiota and growth retardation in mammals. Jiang et al. (2021) identified 11 bacterial species, especially *Prevotella copri*, enriched in low feed efficiency pigs. Nuñez et al. (2025) found that the inclusion of gut microbiota composition in pigs can increase the prediction accuracy of feed efficiency and body weight. Gut microbiome has been identified to contribute to altered metabolism in a pig model of undernutrition (Chang et al., 2021). And the accumulated evidences have indicated that the disruption of “normal” gut microbiota (enteric dysbacteriosis) may contribute to the pathogenesis of undernutrition. For example, the administration of antibiotics may disturb the normal gut microbiota balance of the animals, which may cause growth retardation (Lupindu et al., 2015). The gut microbiota from undernourished donors also ameliorated growth and metabolic abnormalities in recipient animals (Blanton et al., 2016). Gehrig et al. (2019) described the biological features of gut microbiota of gnotobiotic animals and undernourished children that were transitioned from severe acute malnutrition to a state of persistent moderate acute malnutrition accompanying with persistent microbiota immaturity. However, probiotic *Bacillus amyloliquefaciens* C-1 can improve growth performance and regulate the gut microbiota of growth-retarded beef calves (Du et al., 2018).

The effect of gut microbiota on growth phenotypes is mediated through indirect influence on the somatotropic axis. Inflammatory proteins which are stimulated by infection, such as C-reactive protein and alpha-1 acid glycoprotein, are inversely associated with IGF-1 and linear growth (Prendergast et al., 2014). However, germ-free mice showed significantly less weight gain and body length during lactation compared to conventionally raised mice (Schwarzer et al., 2016). This difference became more pronounced following weaning in the presence of a depleted diet and appear to be attributed to the reduced skeletal growth (Schwarzer et al., 2016). IGF-1, a mediator of the effects of growth hormone (GH), was also significantly depleted in germ-free animals, suggesting the essential role of the microbiota in endocrine-mediated growth pathways (Schwarzer et al., 2016). In both *Drosophila* and mice, special strains of *Lactobacillus plantarum* can restore normal growth, IGF-1 production, and activity and sensitivity of peripheral tissue to GH (Storelli et al., 2011; Schwarzer et al., 2016). Recent study also suggested that short-chain fatty acids (SCFAs) restore bone mass, growth, and IGF-1 release in animals showing growth deficits induced by antibiotics (Yan et al., 2016). Hence, microbial fermentation metabolites might play a role in the regulation of somatotropic axis stability and growth phenotypes in early life (Yan et al., 2016). The metabolic disorders of bile acids might relate to the occurrence of chronic diseases, and then affect growth (Sung et al., 2017; Worthmann et al., 2017; Molinaro et al., 2018). For example, Riba et al. (2020) reported that *Giardia lamblia* infection altered the enteric microbiota composition, which leaded to enhanced bile acid deconjugation and increased expression of fibroblast growth factor 15, and finally resulted in elevated energy expenditure, dysregulated lipid metabolism, reduced adipose tissue and body weight gain in the infected mice. However, whether the generation of stunted pigs is related to the dysbacteriosis of gut microbiota remains unknown.

In this study, we collected feces and serum samples from pigs showing growth retardation and their littermates with normal growing speed in two experimental pig cohorts. We first performed 16S rRNA gene and shotgun metagenomic sequencing analyses in fecal samples to identify gut bacterial taxa and function capacities associated with pig growth retardation. We then measured the concentrations of serum metabolites by untargeted metabolome methods and fecal SCFAs to identify the metabolites and SCFAs associated with the growth retardation of pigs and analyze their correlations with the shifts of gut microbiome. The study was to identify the biomarkers significantly associated with pig growth, and provided the knowledge for improving pig growth by regulating gut microbiome.

## Results

### Identification of piglets showing growth retardation in a commercial pig farm

**Figure 1:**
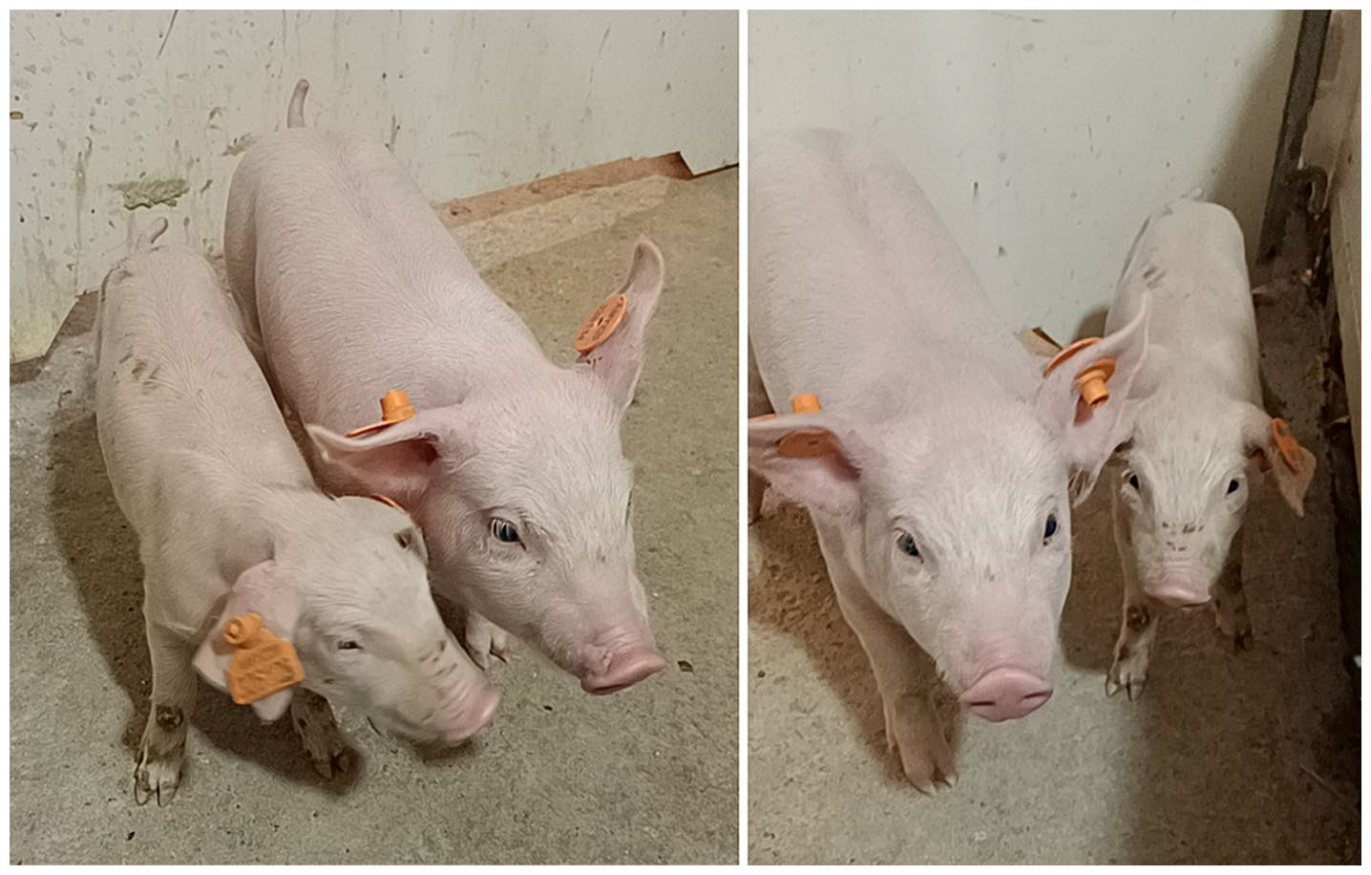
Stunted pigs showing growth retardation compared to its full-sib normal growing pigs.

We phenotyped the growth traits of Large White piglets in a commercial pig farm in July 2017. A total of 28 piglets at the age of 21 days (14 males and 14 females) showing obvious growth retardation (Figure 1) were defined as stunted pigs and treated as a discovery cohort. The piglets showing normal growth and having the same gender were selected from the littermates of stunted pigs (full-sib pairs) and considered as controls. Both feces and serum samples were collected from stunted pigs and their sib-pairs of counterparts. Compared with stunted pigs, their full-sib pairs of normal-growing pigs had significantly higher body weight (*t*-test, *p* < 0.001) and average daily body weight gain (*t*-test, *p* < 0.001) at the time of sampling (Figure 1 and Table 1). However, the body weight at birth was not significantly different between stunted pigs and their counterparts (*t*-test, *p* > 0.05) (Table 1). To further validate the association of gut microbial composition with growth retardation of pigs, we used another 70 pure-breed Large White pigs (35 stunted pigs and 35 full-sibs of normal pigs, 36 female and 34 male) from the same farm as the validation cohort (June 2018). Similar to the findings in the discovery cohort, compared with stunted pigs, the full-sib normal pigs also had significantly higher body weight (*t*-test, *p* < 0.001) and average daily body weight gain (*t*-test, *p* < 0.001) at the sampling time (Table 1). Furthermore, the body weight at birth was not significantly different between stunted pigs and full-sib normal-growing pigs (*t*-test, *p* > 0.05) (Table 1).

**Table 1:** The growth phenotype of the experimental cohorts.

Group	BW^*^ at birth (kg)	BW at sample collection (kg)	Average daily gain (kg)
Discovery cohort			
Stunted pigs (*n* = 28)	1.14 ± 0.36^a^	4.59 ± 0.67^a^	0.09 ± 0.01^a^
Full-sib normal pigs (*n* = 28)	1.32 ± 0.39^a^	7.87 ± 0.74^b^	0.23 ± 0.02^b^
Validated cohort			
Stunted pigs (*n* = 35)	1.13 ± 0.25^a^	3.04 ± 0.44^a^	0.10 ± 0.02^a^
Full-sib normal pigs (*n* = 35)	1.42 ± 0.32^a^	6.23 ± 0.40^b^	0.28 ± 0.03^b^

* BW, body weight. The same superscript letter in the column represents no significant difference, and different superscript letter indicates the difference of body weight achieved significance level.

### Bacterial taxa associated with growth retardation of piglets based on 16S rRNA gene sequencing

The V4 hypervariable region of the 16S rRNA gene was amplified and sequenced for 56 feces samples in the discovery cohort. The amplicon sequencing generated 2,253,635 clean reads (1,27,674 unique sequences). Sequence reads were clustered at 97% of sequence identity, yielding 1,289 operational taxonomic units (OTUs). The α-diversity of gut microbiota was compared between stunted pigs and full-sib counterparts of normal pigs. The stunted pigs had significantly higher observed species, chao, ace, and Simpson indices (Supplementary Figures 1A–D), but significantly lower Shannon index (Supplementary Figure 1E).

**Figure 2:**
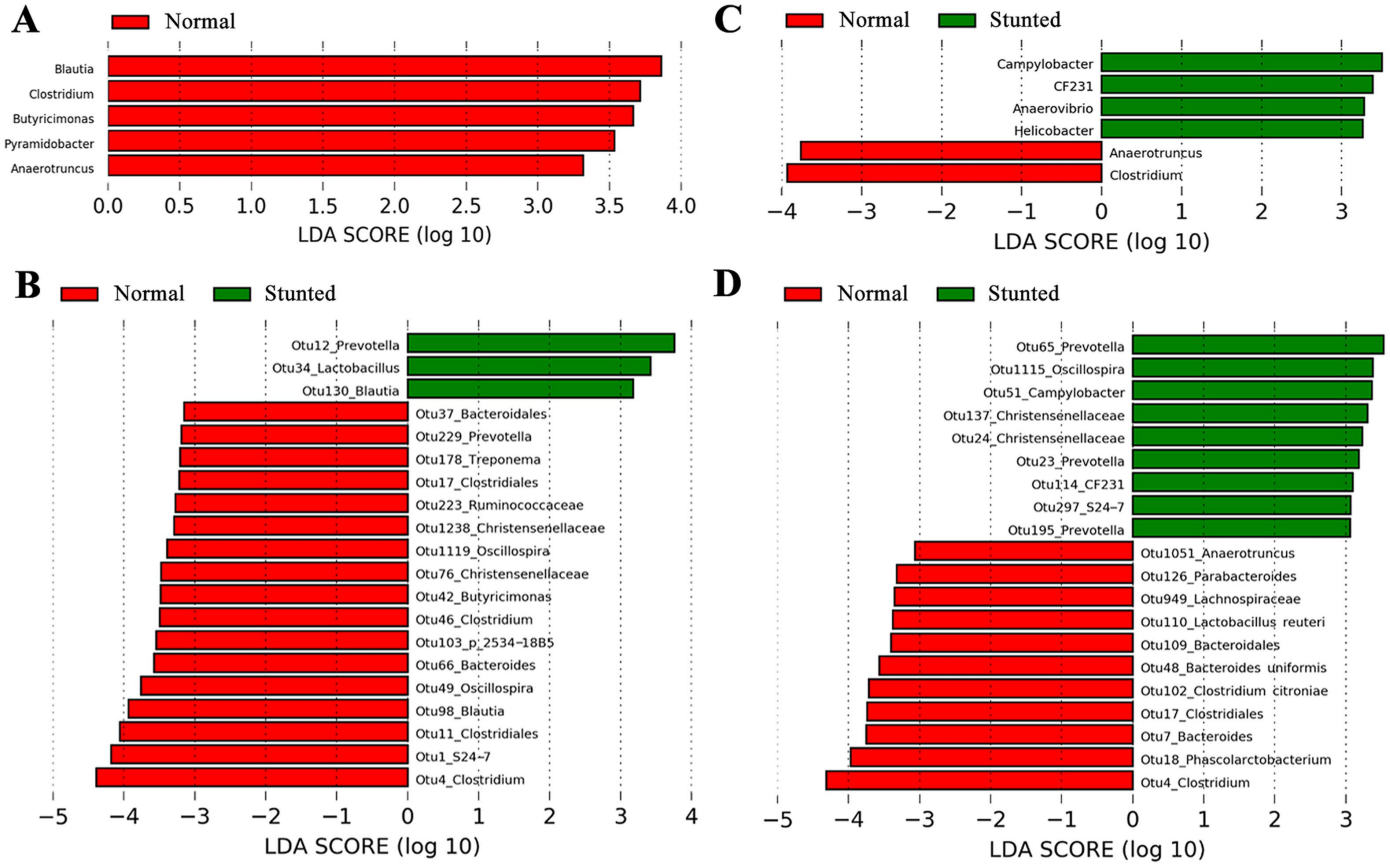
Bacterial genera and OTUs showing significantly different abundances between stunted pigs and full-sib normal growing pigs in the discovery and validation cohorts by 16S rRNA gene sequencing. **(A)** Differential bacterial genus identified in the discovery cohort. **(B)** Parts of differential OTUs (top 20 OTUs showing significantly different abundances) in the discovery cohort. **(C)** Differential bacterial genera identified in the validation cohort. **(D)** Parts of differential OTUs (top 20 OTUs showing significantly different abundances) in the validation cohort. A linear discriminant analysis (LDA) effect size (LEfSe) analysis was used to identify differential taxa. LDA score ≥2 was set as the threshold.

We further compared the bacterial compositions of gut microbiota between stunted pigs and full-sib pairs of normal pigs to identify bacterial taxa associated with growth retardation. At the genus level, five genera were significantly enriched in normal pigs (Figure 2A), including *Blautia*, *Clostridium*, *Butyricimonas*, *Pyramidobacter*, and *Anaerotruncus*. At the OTU level, 39 OTUs showed significantly different abundances in the gut between stunted pigs and normally growing pigs (Figure 2B and Supplementary Table 1), including six OTUs enriched in stunted pigs and 33 OTUs enriched in normal pigs. The 33 OTUs enriched in normal pigs were mostly annotated to *Clostridiales*. The OTU4 annotated to *Clostridium* showed the most significant enrichment in normal pigs.

The V4 hypervariable region of the 16S rRNA gene was also sequenced for 70 feces samples in the validation cohort, and similar results were obtained. Stunted pigs had significantly higher observed species, chao, ace, and Simpson (Supplementary Figures 2A–D), but lower Shannon index (Supplementary Figure 2E). At the genus and OTU level, we found six genera and 63 OTUs showing significantly different abundances between stunted pigs and full-sib counterparts of normal pigs (Figures 2C,D and Supplementary Table 2). Interestingly, *Clostridium* and *Anaerotruncus* were enriched in normal pigs in both discovery and validation cohorts, and the OTU4 (annotation to *Clostridium*) showed the most significant enrichment in both cohorts.

### Identification of bacterial species and function capacity associated with growth retardation in pigs by metagenomic sequencing

To further identify bacterial species and functional capacity of the gut microbiome associated with growth retardation of pigs, we performed shotgun metagenomic sequencing of 32 fecal samples, including 12 samples from the discovery cohort (six samples from each of stunted and full-sib normal groups) and 20 samples from the validation cohort (10 stunted pigs and 10 full-sib normal counterparts). The sequence assembly of 32 samples obtained 5,368,402 contigs with an average length of 1,847 bp and an average N50 length of 3,879 bp (Supplementary Table 3). The phylogenetic composition of the fecal microbiota was determined by blasting the contigs against the NCBI nucleotide (NR) database. Similar to the results obtained from 16S rRNA gene sequencing data, *Prevotella* and *Bacteroides* were the two most abundant genera of gut microbiota. At the species level, a total of 457 bacterial species were detected in all 32 samples. *Bacteroides fragilis* was the most abundant bacterial species in the tested samples.

**Figure 3:**
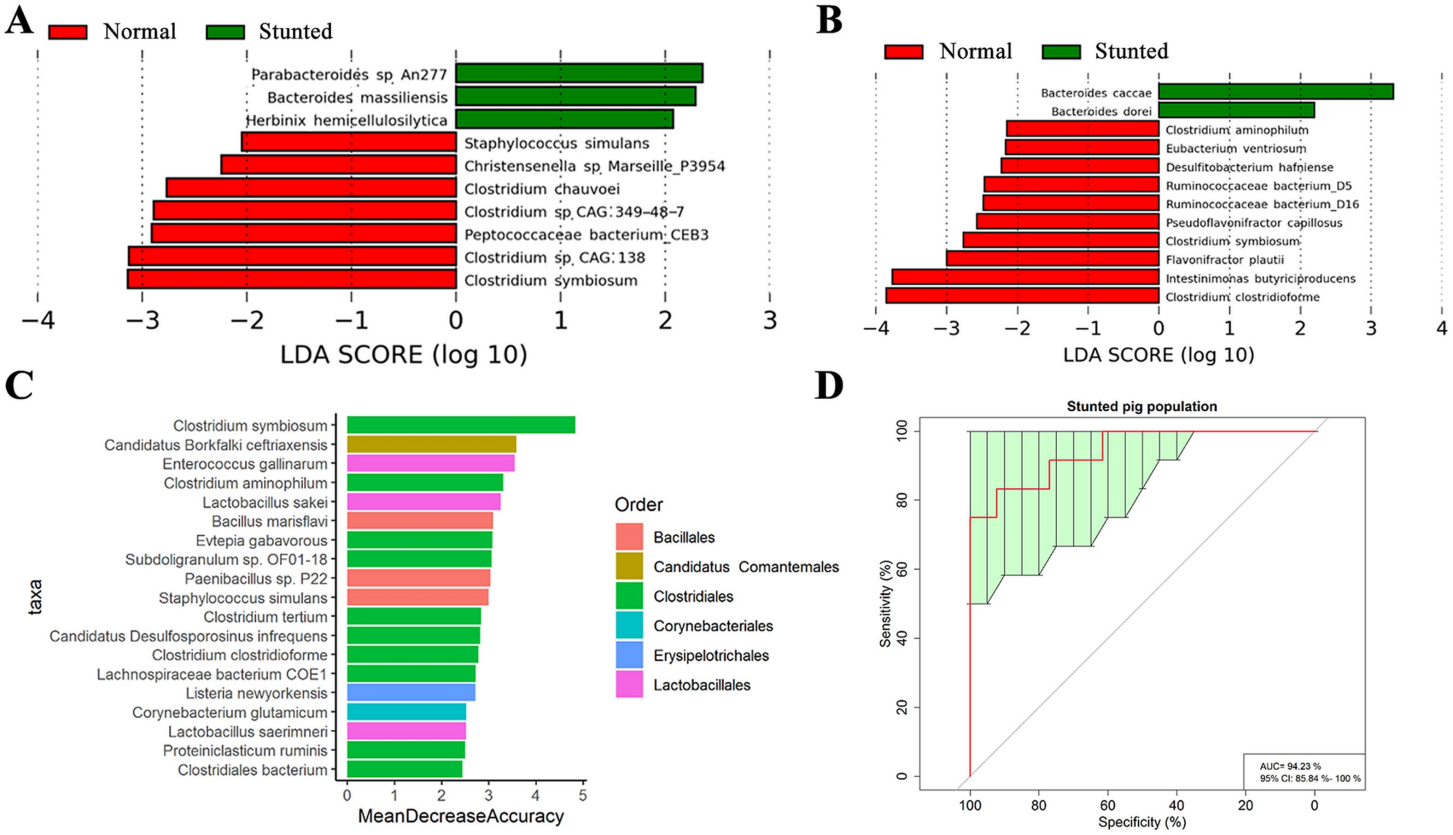
Bacterial species associated with growth retardation of pigs based on metagenomic sequencing data. **(A,B)** Bacterial species showing significantly different abundances between stunted pigs and full-sib normal growing pigs in the discovery **(A)** and validation cohorts **(B)**. LEfSe analysis was used to identify differential bacterial species. LDA score ≥2 was set as the threshold. **(C)** Bacterial species that could discriminate stunted pigs and normal growing pigs by random forest model. **(D)** Receiver operating curve (ROC). The AUC was 94.23% with the 95% CI of 85.84–100%.

The β-diversity analysis based on metagenomic sequencing data showed significant difference in the gut microbiota compositions between stunted pigs and normal full-sib littermates in both discovery and validation cohorts (Supplementary Figure 3). We further identified bacterial species associated with growth retardation. A total of 10 bacterial species had different abundances between stunted pigs and full-sib normal counterparts in the discovery cohort (Figure 3A), including three species significantly enriched in stunted pigs and seven species having higher abundance in normal pigs. The bacteria enriched in the gut of full-sib littermates showing normal growth mainly belonged to *Clostridium* (4/7 species). In the validation cohort, we identified 12 bacterial species having distinct enrichments between stunted pigs and full-sib normal pigs. Among them, two *Bacteroides* spp. were enriched in the gut microbiome of stunted pigs, whereas three *Clostridium* spp. were enriched in normal growing full-sib littermates (Figure 3B). The LEfSe analysis combining all 32 metagenomic sequencing data identified 21 bacterial species showing differential abundances between stunted and normal pigs, including two species enriched in stunted pigs and 19 species enriched in normal sib-pairs (Supplementary Figure 4). Interestingly, *Clostridium symbiosum* was the only bacterial species showing higher abundances in normal-growing pigs in all the discovery and validation cohorts, and combined dataset. Subsequently, a random forest analysis was performed to identify bacterial biomarkers for discriminating stunted pigs from full-sib counterparts of normal pigs based on metagenomic sequencing data in the discovery cohort (Supplementary Figure 5A), validation cohort (Supplementary Figure 5B), and combined cohort of all 32 samples at the species level (Figure 3C). The results also showed that *Clostridium symbiosum* was the most distinct bacterial marker distinguishing stunted pigs and normally growing pigs with robust and high diagnostic accuracy of the area under the curve (AUC) 92.00, 93.06 and 94.23% (Supplementary Figures 5C,D and Figure 3D).

**Figure 4:**
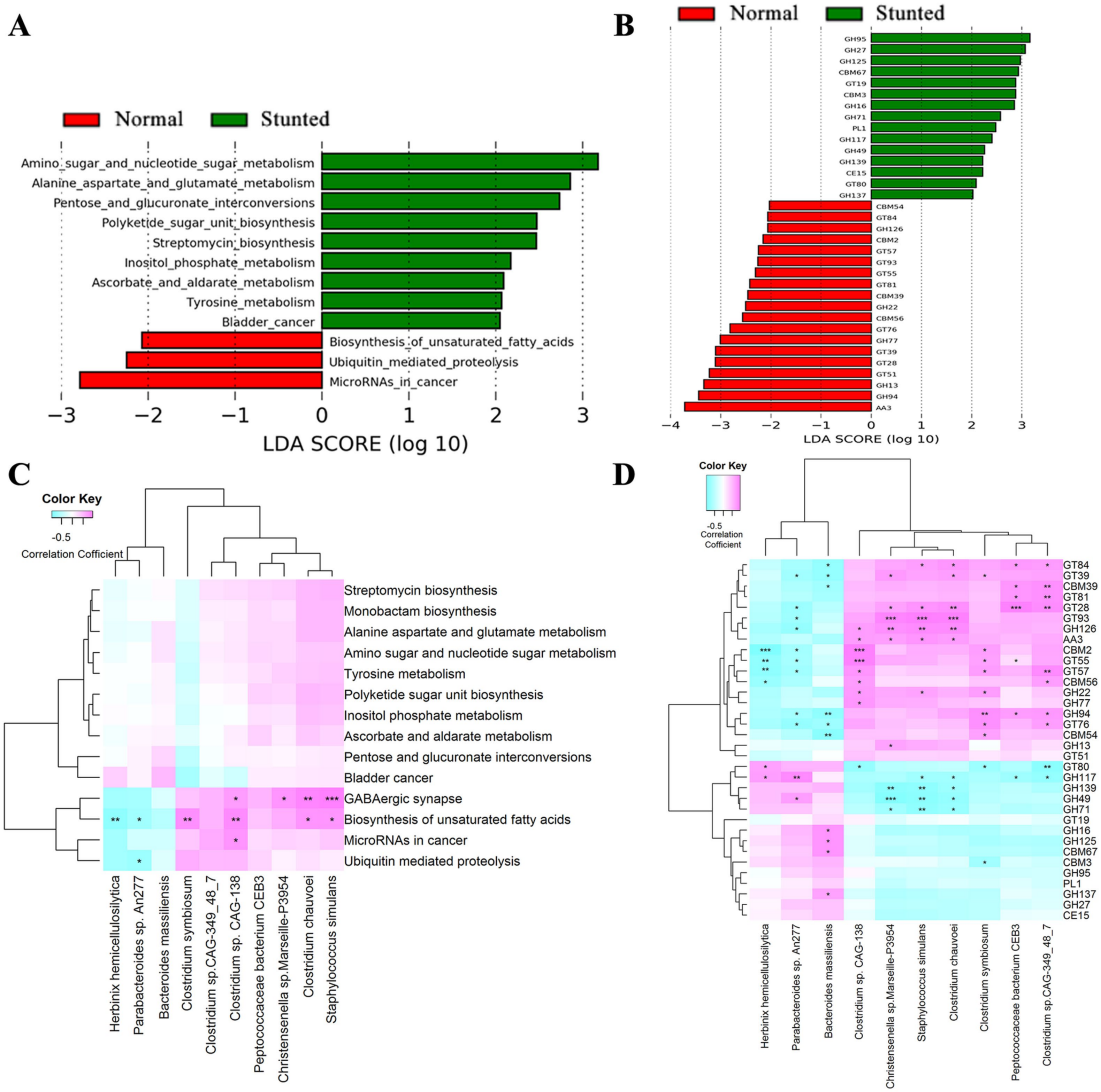
KEGG pathways and CAZymes showing significantly different abundances between stunted pigs and normal growing pigs, and their relationships with differential bacterial species in the discovery cohort. **(A)** Differential KEGG pathways. **(B)** Differential CAZymes. **(C,D)** The heat maps showing the relationships between differential bacterial species and differential KEGG pathways **(C)** and CAZymes **(D)**. The *X*-axis represents the bacterial species. The *Y*-axis indicates the differential KEGG pathways/CAZymes. ^*^p < 0.05, ^**^p < 0.01, and ^***^p < 0.001.

We then compared the potential functional capacities of the gut microbiome between stunted and normal-growing pigs by aligning and classifying the microbial genes to the KEGG and Carbohydrate-Active enZYmes (CAZy) databases. We identified 12 and 9 KEGG pathways showing distinct enrichments between stunted pigs and their full-sib normal pairs in the discovery (Figure 4A) and validation cohorts (Supplementary Figure 6A). The pathway of biosynthesis of unsaturated fatty acids was the only differential function term identified in both the discovery and validation cohorts. For CAZymes, a total of 34 and 14 CAZymes had significantly different abundances between stunted pigs and full-sib normal pigs in the discovery (Figure 4B) and validation cohorts (Supplementary Figure 6B), including PL1 (pectate lyase) and GT39 (protein alpha-mannosyltransferase) that were identified in both discovery and validation cohorts.

We further evaluated the correlation between the shifts in bacterial species and the changes in KEGG pathways and CAZymes using a Spearman correlation analysis. The results indicated that *Clostridium symbiosum* was positively and significantly correlated with the functional terms of biosynthesis of unsaturated fatty acids in both discovery (Figure 4C) and validation cohorts (Supplementary Figure 6C). About the CAZymes, we found that the GT39 was positively and significantly correlated with *Clostridium symbiosum* in both discovery (Figure 4D) and validation cohorts (Supplementary Figure 6D). These results suggested the contribution of the changes of bacteria species to the shifts of KEGG pathways and CAZymes.

### Differential serum metabolite profiles between stunted pigs and full-sib normal pigs

**Figure 5:**
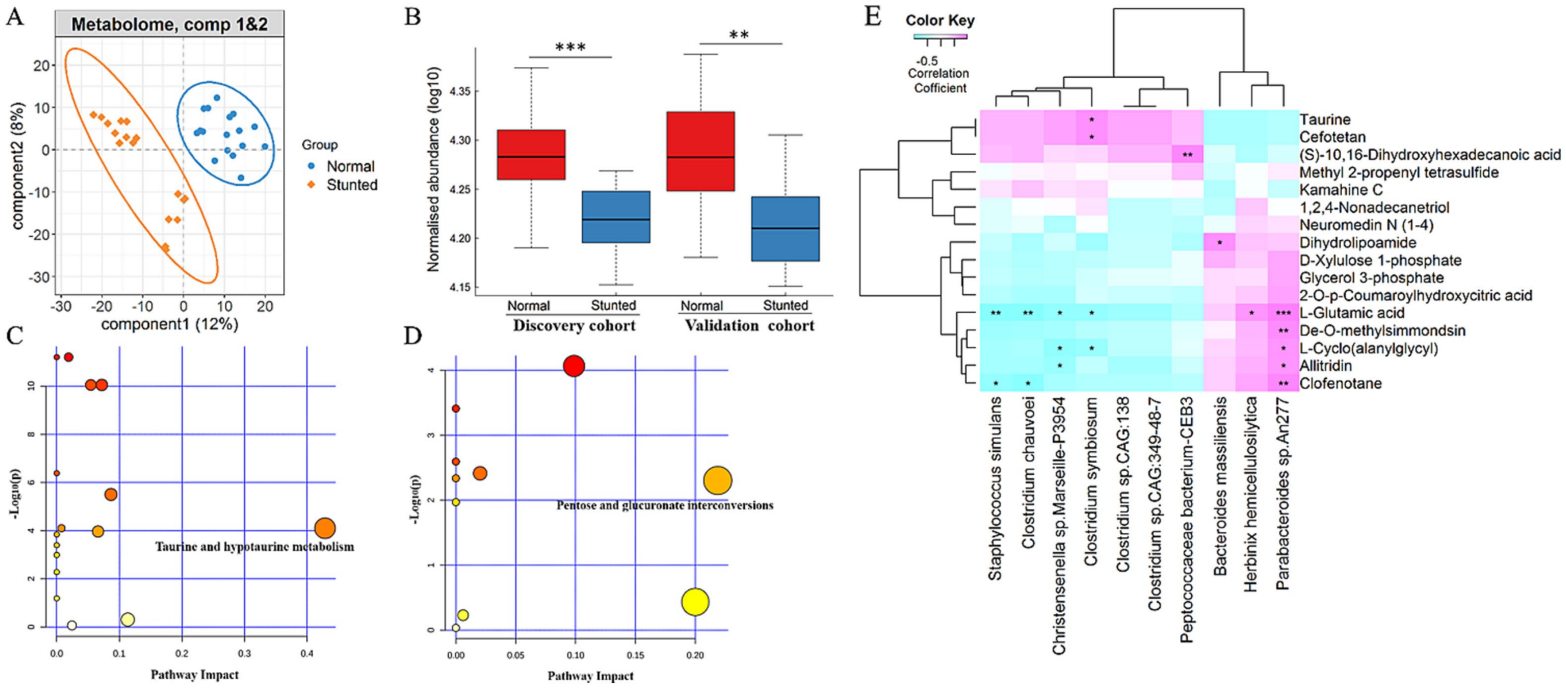
The changes in serum metabolome between stunted pigs and normal growing pigs, and the pathways enriched by differential serum metabolite features. **(A)** sPLS-DA plot of serum metabolite profiles indicating the significant difference in serum metabolite profiles between stunted pigs and full-sib normal pigs in the discovery cohort. **(B)** Significantly different abundances of serum taurine in both discovery and validation cohorts. **(C,D)** KEGG pathways enriched by the metabolite features showing higher abundances in full-sib normal pigs **(C)** and stunted pigs **(D)**. The *X*-axis and the size of the dots indicate the pathway impact of altered metabolite features, and the *Y*-axis shows the *p*-value obtained for each pathway in the enrichment analysis. The size and color of the dots indicate the overall pathway impact. **(E)** The relationships of altered serum metabolite features with differential bacterial species in the discovery cohort. The *X*-axis represents bacterial species. The *Y*-axis represents the differential serum metabolite features.

To systematically evaluate the shifts of serum metabolome between stunted pigs and their full-sib normal-growth pairs, untargeted serum metabolomic profiles were determined using UPLC-MS. After normalization, we obtained 1,408 metabolites in the discovery cohort. An obvious shift in the global metabolome was observed between stunted pigs and their full-sib normal pigs in the discovery cohort (Figure 5A). Specifically, we identified a total of 99 metabolite features showing distinct enrichment patterns between stunted pigs and full-sib normal pigs (Supplementary Table 4). The same analysis was also performed for serum metabolites in the validation cohort. A total of 2,740 metabolites were obtained after normalization. Similar to the results obtained in the discovery cohort, stunted pigs showed an obvious shift in the global metabolome (Supplementary Figure 7A), and a total of 409 metabolite features were identified as differential metabolites between stunted and normal pigs (Supplementary Table 5). Interestingly, a total of 16 differential metabolite features were identified in both discovery and validation cohorts, including taurine, (S)-10, 16-dihydroxyhexadecanoic acid, and L-glutamic acid (Figure 5B and Supplementary Table 6). The differential metabolite features identified in stunted pigs and normal-growth littermates were annotated and classified using KEGG pathways separately. We found that the metabolites having higher abundances in normal littermates were significantly enriched in the KEGG pathway of taurine and hypotaurine metabolism (Figure 5C), while the pathway of pentose and glucuronate interconversions was enriched by the metabolites having higher abundances in stunted pigs (Figure 5D). To further evaluate the correlations between differential bacterial species and metabolite features, a Spearman correlation analysis was performed independently in the discovery and validation cohorts. The results indicated that *Clostridium symbiosum* was positively and significantly correlated with taurine in both discovery and validation cohorts (Figure 5E; Supplementary Figure 7B).

**Figure 6:**
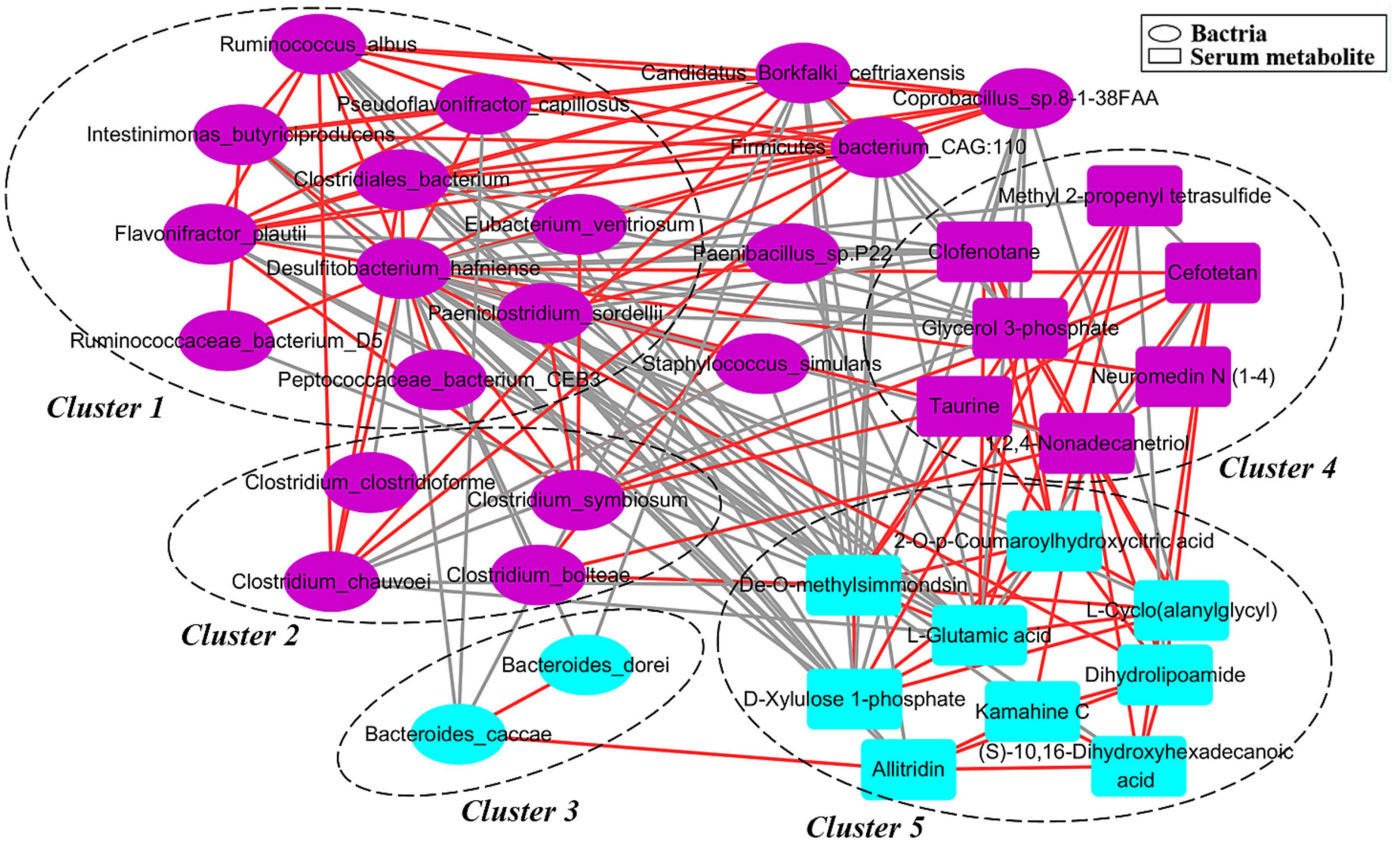
A co-occurrence network constructed with bacterial species and serum metabolites showing different abundances between stunted pigs and normal growing pigs. Purple and blue dots indicate the differential bacterial species and serum metabolites, respectively. Edges between nodes indicate Spearman’s negative (light gray) or positive (light red) correlation.

We further explored the potential correlations between differential bacterial species and serum metabolites by co-occurrence network analysis. Strong and broad co-occurring relationships were observed between each other (Figure 6). The differential bacterial species and serum metabolites were mainly aggregated into five clusters in this network. The bacterial species belonging to clusters 1 and 2 were enriched in full-sib normal pigs. And the bacterial species enriched in stunted pigs were mainly included in the cluster 3. Interestingly, the *Clostridium symbiosum* was positively and significantly correlated with the bacterial species and serum taurine enriched in normal pigs.

### Comparison of fecal SCFAs between stunted pigs and full-sib normal pigs

**Figure 7:**
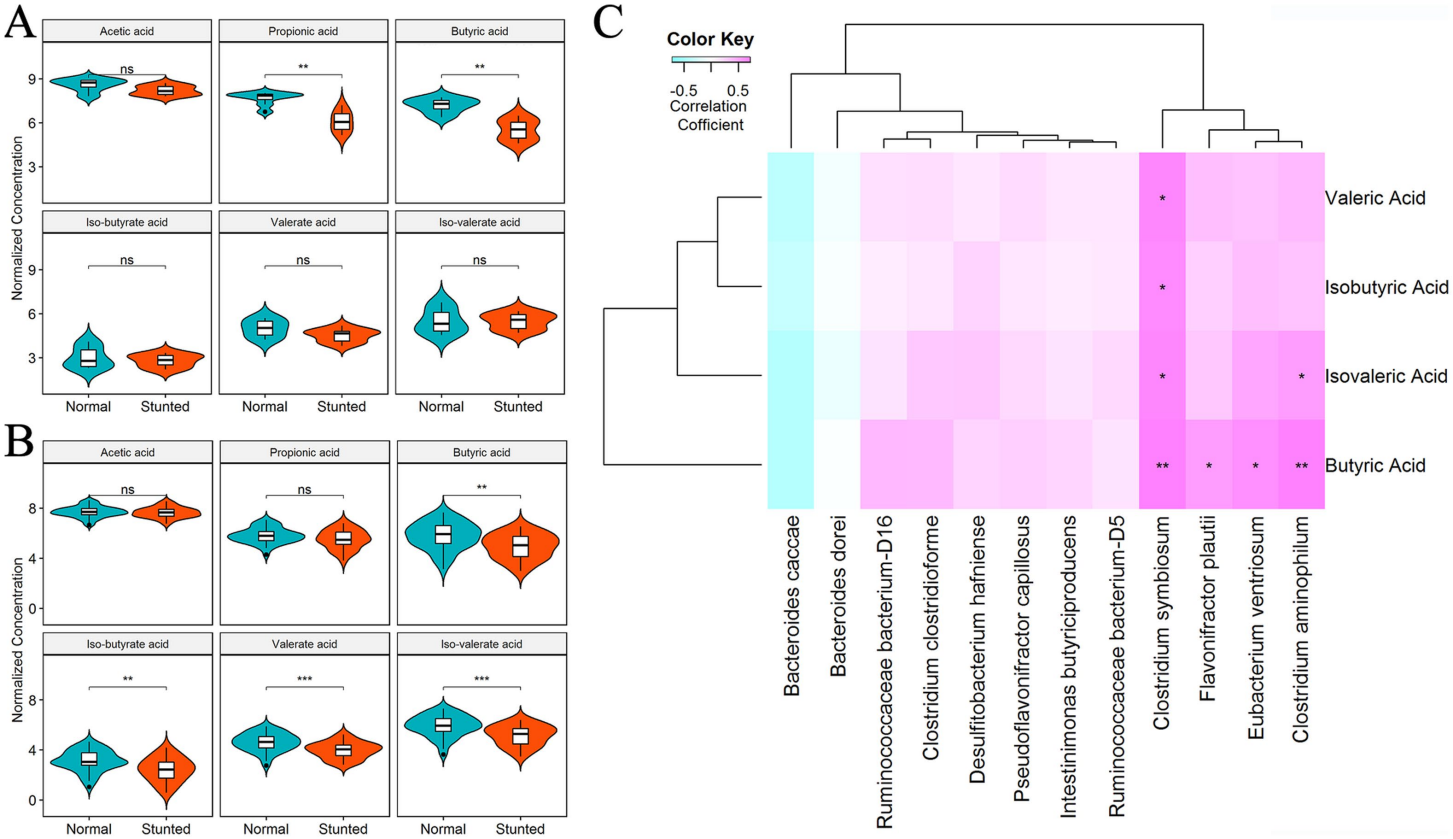
Comparison of the concentrations of fecal short chain fatty acids (SCFAs) between stunted pigs and full-sib normal growing pigs. **(A,B)** Comparison of the concentrations of fecal SCFAs in the discovery **(A)** and validation cohort **(B)**. **(C)** The heat map showing the relationship between differential bacterial species and SCFAs in the validation cohort. The *X*-axis shows bacterial species. The *Y*-axis indicates SCFAs.

As many bacterial species enriched in normal growing pigs are related to the production of SCFAs (e.g., *Clostridium*), we measured the concentrations of fecal SCFAs (acetic acid, propionic acid, butyric acid, iso-butyrate acid, valerate acid, and iso-valerate acid) in both discovery and validation cohorts. In the discovery cohort, propionic acid and butyric acid showed higher concentrations in normal-growing pigs (Figure 7A). In the validation cohort, normal-growing pigs had higher abundances of butyric acid, iso-butyrate acid, valerate acid, and iso-valerate acid (Figure 7B). Butyric acid was the SCFA having higher abundances in normal growing pigs in both discovery and validation cohorts. Because the samples having the metagenomic sequencing data were not measured SCFAs in the discovery cohort, we only analyzed the relationship between differential bacterial species and SCFAs in the validation cohort using Spearman correlation analysis. As we expected, the results indicated that *Clostridium symbiosum* was positively and most significantly correlated with the concentration of fecal butyric acid (Figure 7C).

## Discussion

Gut microbiota has been treated as an important organ of the body. It takes part in many physiological processes, such as metabolism, immunity, and even behaviors (Davidson et al., 2020; Ruff et al., 2020; Wu et al., 2020). Alterations of gut microbiota may related to growth retardation in some agricultral livestock, such as pigs and cattle (Du et al., 2018; Jiang et al., 2021). Several studies have also identified significant associations between gut microbiota and malnutrition in children (Blanton et al., 2016; Vonaesch et al., 2018; Gehrig et al., 2019). Growth retardation (stunted pigs) has brought a big economic loss to the pig industry. However, to our knowledge, there was no study reporting the association between gut microbiota and growth retardation in pigs. In this study, we compared the bacterial composition of the gut microbiome between stunted pigs and their full-sib normal littermates by 16S rRNA gene and metagenomic sequencing, and identified bacterial species associated with growth retardation. We found that the changes in gut microbiome were correlated with the shifts of serum metabolome and fecal SCFAs, and then, should result in the growth retardation of pigs. To our knowledge, this is the first study evaluating the relationships of the composition and functional capacity of the gut microbiome with growth retardation of pigs.

The stunted pigs had significantly higher richness (higher observed species, chao, and ace), but significantly lower diversity and evenness of gut microbiota (higher Simpson index and lower Shannon index) in both discovery and validation cohorts. This indicated that a more diverse gut microbiome could help maintain host resilience, resistance, and stability under environmental stresses (Konopka, 2009). The present study also illustrated that the gut microbiome composition changed significantly between stunted pigs and normal littermates. *Clostridium* was significantly enriched in normal pigs. *Clostridium* can produce SCFAs or bioactive substances that play important roles in gut homeostasis and disease resistance (Gilbert, 1976; Breyner et al., 2017; Zhou et al., 2017). The OTU4 most significantly enriched in normal pigs was also annotated to *Clostridium*. The OTUs that were assigned to *Clostridiales* were associated with the production of butyric acid in the gut. Butyrate can provide an energy source for intestinal epithelial cells (Hamer et al., 2008).

*Clostridium symbiosum* was the only bacterial species showing different abundances between stunted pigs and normal-growing pigs in both discovery and validation cohorts. Random forest analysis also observed a robust and high diagnostic AUC for distinguishing stunted pigs using *Clostridium symbiosum*. These results suggested that *Clostridium symbiosum* was a key bacterial biomarker related to pig growth. Many studies have suggested that *Clostridium symbiosum* could ameliorate growth and metabolic abnormalities in stunted pigs and recipient animals (Blanton et al., 2016; Gehrig et al., 2019). Furthermore, functional capacity analysis revealed that *Clostridium symbiosum* was significantly and positively associated with KEGG pathways related to the biosynthesis of unsaturated fatty acids. Biosynthesis of unsaturated fatty acids was important and necessary for the early stages of development (Nakamura et al., 2001). It could influence growth, metabolism, and immunity in infancy and childhood (Gibson et al., 2011; Perng et al., 2015; Brzezinska et al., 2016).

Serum metabolome analysis found that the concentration of serum taurine was significantly different between stunted pigs and full-sib normal pigs in both discovery and validation cohorts. Of note, in rodents, taurine is predominantly conjugated with bile acids (BAs) (Ahmad and Haeusler, 2019). Patients with type 2 diabetes mellitus and Crohn’s disease have been reported to have increased concentrations of taurine-conjugated BA species (Linnet and Mertz Nielsen, 1983; Wewalka et al., 2014). Furthermore, the differential metabolites were significantly enriched in the KEGG pathways of taurine and hypotaurine metabolism. Taurine and hypotaurine metabolism include the synthesis of primary bile acids in the liver from cholesterol and conjugation with taurine before excretion (Molinaro et al., 2018). Many studies have found that gut microbes can deconjugate primary bile acids and transform them into secondary bile acids (Worthmann et al., 2017; Molinaro et al., 2018). Especially, Joyce et al. (2014) demonstrated that the gut microbiota regulated the host weight gain and lipid metabolism through bile acid modification by bacteria (Joyce et al., 2014). In this study, we also found that *Clostridium symbiosum* was significantly and positively correlated with the concentration of serum taurine. This suggested that *Clostridium symbiosum* may improve pig growth through taurine and hypotaurine metabolism.

SCFAs are produced mainly through anaerobic fermentation of carbohydrates that cannot be digested and absorbed in the small intestine. The pathways of SCFA production are relatively well understood (Miller and Wolin, 1996) and described in detail (Flint et al., 2015). In this study, we found that butyric acid was the only differential SCFA identified in both discovery and validation cohorts. Butyrate is a favorable energy source, playing important roles in gut homeostasis and disease resistance (Clausen and Mortensen, 1995; Donohoe et al., 2011; den Besten et al., 2013; van der Beek et al., 2015; Wang et al., 2020). Recent data also suggests that butyrate suppresses TNF-α, IL-6, and myeloperoxidase activity by preventing NF-kB activation in Kupffer cells (Qiao et al., 2014). Furthermore, the Spearman correlation analysis indicated that *Clostridium symbiosum* was positively and most significantly correlated with butyric acid in the validation cohort. A shift toward the butyrate-producing organism *Clostridium symbiosum* along with increased butyrogenesis was observed in low-fat and high-fiber feeding in humans (Holmes et al., 2012). However, all these changes can translate into long-term impacts on host metabolism.

## Conclusion

In summary, we found that the composition and functional capacity of the fecal microbiome, fecal SCFAs, and serum metabolites were significantly changed between stunted pigs and their corresponding full-sibs showing normal growth. The *Clostridium symbiosum* was identified as a key bacterial species associated with pig growth. It was significantly correlated with the changes in the functional capacity of unsaturated fatty acid biosynthesis in the gut microbiome. Untargeted metabolome analysis identified that the concentration of serum taurine was associated with pig growth retardation and correlated with the abundance of *Clostridium symbiosum* in the gut microbiome. All results suggested that gut microbes, such as *Clostridium symbiosum* may affect pig growth by changing the metabolites (e.g., taurine and butyric acid). This study gave important insights into the effect of the gut microbiome on pig growth performance through their metabolites and provided the theoretical basis for improving growth of stunted pigs by modulating the gut microbiome. However, the results in this study were only based on the association studies, and the causality and underlying mechanisms would need to be further addressed in future studies.

## Materials and methods

### Experimental animals and sample collection

Two experimental pig cohorts were used in this study. The discovery cohort was comprised of 56 commercial pure-breed Large White pigs (28 female and 28 male) including 28 stunted pigs and 28 full-sib normal growth pigs from Liangyeshan farm (Fujian). Fecal samples were collected in July 2017. The validation cohort contained 70 pure-breed Large White pigs (36 female and 34 male) from the same farm, including 35 stunted pigs and 35 full-sib normal growth pigs. Their fecal samples were collected in June 2018. The feeding and management patterns were similar for two piglet cohorts. Briefly, all piglets stayed with their mothers during the suckling period and allowed to nurse freely until weaning at 21 days of age. Clean water was provided *ad libitum*. Fresh feces from all piglets were collected from each animal’s anus before weaning near the age of 21 days. All pigs were healthy and received no probiotic or antibiotic therapy during the period from birth to sampling. Fecal samples were immediately snap-frozen in liquid nitrogen for transportation, and then stored at −80 °C until use. Blood samples were also collected from each experimental pig at the time of sampling.

### Microbial DNA extraction and 16S rRNA gene sequencing

Microbial DNA was extracted from 126 fecal samples including 56 samples from the discovery cohort and 70 samples from the validation cohort using the QIAamp Fast DNA Stool Mini Kit (Qiagen, Germany) according to the manufacturer’s instructions. The concentration of DNA was determined using a Nanodrop-1000 (Thermo Scientific, United States) and the DNA purity was confirmed by 0.8% (w/v) agarose gel electrophoresis. All DNA samples were stored at −20 °C until use.

The V4 hypervariable region of the 16S ribosomal RNA (rRNA) gene was amplified for all 126 fecal samples using conserved primers 515F (5′-GTGCCAGCMGCCGCGGTAA) and 806R (5′-GGACTA CHVGGGTWTCTAAT). The parameters for PCR amplification were as follows: 2 min initial denaturation at 95 °C, 30 cycles of denaturation at 95 °C for 30 s, annealing at 55 °C for 30 s, elongation at 72 °C for 30 s, with a final extension step at 72 °C for 5 min. After purification, the amplicons of each sample were constructed the libraries and sequenced on an Illumina MiSeq platform (Illumina, United States).

### Taxonomic classification and diversity analysis

The raw data were filtered to eliminate adapter sequences and low-quality reads (Looft et al., 2012). Paired-end clean sequence reads were then assembled into tags using FLASH software (Alain et al., 2014). To avoid statistical bias caused by uneven sequencing depth, the sequencing depth for each sample was rarefied to 10,000 tags. Tags were clustered into operational taxonomic units (OTUs) at 97% sequence identify using USEARCH software (v7.0.1090) (Thompson et al., 2008) and the UPARSE-OTU algorithm. OTU taxonomic assignments based on the 16S rRNA gene sequences were performed using the RDP classifier program (v2.2) (Pluske, 2013). The α-diversity indexes including observed species and Shannon index were measured using Mothur software (Niu et al., 2015), and visualized by ggplot2 (v. 4.0.0). To identify bacterial taxa showing differential abundance between stunted and normal growth pigs, a linear discriminant analysis (LDA) effect size (LEfSe) analysis was performed under the condition *α* = 0.01, with an LDA score of at least 2.0 (Ibba and Soll, 2000). False discovery rate (FDR) was used to correct the multiple tests.

### Metagenomic sequencing analysis

A total of 32 fecal samples were used for metagenomic sequencing, including 12 samples (six from stunted pigs and the other six from full-sib normal littermates, half for each sex) from the discovery cohort, and 20 samples from 10 stunted pigs and 10 full-sib pairs showing normal growth in the validation cohort (half for each sex). Sequencing libraries were generated using NEB Next^®^ Ultra^™^ DNA Library Prep Kit (NEB, United States) following manufacturer’s recommendations with an insert size of 350 base pairs (bp), and index codes were added to attribute sequences of each sample. The libraries were analyzed for size distribution using an Agilent 2100 Bioanalyzer, quantified using real-time PCR, and sequenced on a Novaseq 6000 platform (Illumina, United States) with pair-end 150-bp strategy.

The raw sequencing data was preprocessed using Readfq to acquire clean data for subsequent analysis. The clean sequence reads were further blasted against the pig reference genome (Sscrofa 11.1) using Bowtie (v2.2.4) software (Zhao et al., 2015) to filter out host genomic sequencing pollution. Then, the clean data was assembled using MEGAHIT (v1.1.3) (Li et al., 2016). The contigs with more than 500 bp in length were used to predict open reading frames (ORF) using MetaGeneMark (v2.10) (Mach et al., 2015; Vo et al., 2017). CD-HIT (Hamer et al., 2008) software was used to obtain the unique gene catalog. The genes for which the number of reads was less than two in each of all tested samples were filtered using Bowtie (v2.2.4) (Langmead and Salzberg, 2012). The filtered gene catalog was eventually used for all subsequent analysis. DIAMOND (De Filippis et al., 2019) software was used to annotate taxa by blasting the Unigenes to the sequences of bacteria, fungi, archaea and viruses with *e*-values ≤1 × 10^−5^, all of which were extracted from the NCBI NR database. Functional annotations were performed by aligning the putative amino acid sequences which were translated from the predicted genes to KEGG (Arroyo et al., 2016) and CAZy (Reitzer et al., 1979) database. The β-diversity of gut microbiota based on the Bray–Curtis distance was analyzed with metagenomic sequencing data using the q2-diversity plugin in QIIME2. LEfSe analysis was used to identify KEGG pathways and CAZymes enriched in each of normal and stunted pig groups under the threshold of an LDA score >2.0 (Ibba and Soll, 2000). To evaluate the contribution of bacterial species, a random forest classification model was constructed based on bacterial species from metagenomic sequencing data using *k*-fold cross-validation, where *k* represents the number of samples. The efficiency of classification was assessed using receiver operating characteristic (ROC) curves, and the area under the ROC curve (AUC) was measured using the pROC package for R software (V.4.1.2).

### Determination of metabolomic profiles of serum samples

A total of 96 serum samples were used for untargeted metabolomic analysis by an ultra-performance liquid chromatography coupled with quadrupole time-of-flight mass spectrometry (UPLCQTOF/MS), including 34 samples (17 for each of stunted and normal growing pigs) from the discovery cohort, and 62 serum samples (31 samples for each of stunted and normal pigs). All serum samples were thawed on ice and precipitated with pre-cooled methanol (Merck Corp., Germany) as described previously (Liu et al., 2017). Briefly, 300 μL of cooled methanol was added to 100 μL serum. The mixture was vortexed for 1 min, incubated at −20 °C for 20 min, and centrifuged at 15,000 × g (rcf) for 15 min at 4 °C. Supernatant was removed into a clean tube and dried in a Savant vacuum evaporator. Dried supernatant was resolved in 150 μL water: methanol (85.15% v/v) and transferred into the sampling vials pending UPLC-QTOF/MS (Waters Corp., United States) analysis (Dunn et al., 2011). In addition, the pooled quality control (QC) sample was prepared by combining aliquots of equal volume of each tested sample.

Working solution (1.0 μL) was injected into a 100 mm × 2.1 mm, 1.7 mm BEH C18 column (Waters Corp., United States). The pooled QC sample was injected eight times at the beginning of the run to ensure system equilibrium, and then one time for each of 12 samples to further monitor analytical stability. For the positive electrospray ion mode (ES^+^), serum samples were eluted using a linear gradient from 100% A to 100% B (A, water + 0.1% formic acid; B, acetonitrile) at a flow rate of 0.3 mL/min and a column temperature of 40 °C for 22 min. For the negative electrospray ion mode (ES^−^), injected serum samples were eluted on a linear gradient of 100% A to 100% B (A, water + 0.1% formic acid; B, acetonitrile) at a flow rate of 0.3 mL/min at 40 °C of the column temperature for 18 min.

Mass spectrometric data was collected using a Waters Q-TOF Premier (Waters Corp., United States) equipped with an electrospray source operating in either ES^+^ or ES^−^. The source temperature was set at 120 °C, and the desolation gas temperature was set at 350 °C. The capillary voltage was set at 3.0 and 2.5 kV for ES^+^ and ES^−^, respectively. Centroid data were collected from 50 to 1,200 *m*/*z* with a scan time of 0.3 s and interscan delay of 0.02 s. MassLynx software (Waters Corp., United States) was used for system control and data acquisition. Leucine enkephalin was used as the lock mass (*m*/*z* 556.2771 in ES^+^ and 554.2615 in ES^−^) at a concentration of 100 ng/mL and a flow rate of 5 μL/min for all analyses. Progenesis QI software (v2.0) (Nonlinear Dynamics, United Kingdom) was used for feature alignment, non-targeted signal detection and signal integration.

MetaScope embedded in the Progenesis QI (Rusilowicz et al., 2016) was used to annotate the metabolites not only based on neutral mass, isotope distribution and retention time, but also based on the collisional cross-sectional area and MS/MS fragmentation data in the HMDB database. Ion intensity of each peak was obtained and a 3D-matrix containing arbitrarily assigned peak indices (retention time-*m*/*z* pairs), ion intensities (variables) and sample names (observations) was generated. Raw matrices were further filtered by removing peaks with missing values (ion intensity = 0) in more than 80% of the samples and 50% of the QC samples. Each retained peak was then normalized to the QC sample using support vector regression (SVR) from the R package MetNormalizer (Shen et al., 2016) to ensure a high quality of data within an analytical run. The relative standard deviation (RSD) values of the metabolites in the QC samples were set at a threshold of 30% to assess the repeatability of metabolomic data sets.

### Determination of the SCFAs of porcine fecal samples

A total of 88 fecal samples were used for determining the concentration of fecal SCFAs, including 18 samples (nine for each of stunted and normal growing pigs) from the discovery cohort, and 70 fecal samples (35 samples for each of stunted and normal growing pigs). All fresh fecal samples (about 0.3 g/sample) were homogenized in sterile deionized water (1 mg feces/5 μL ddH_2_O, 20%, w/v), then centrifuged at 5,000 rpm, 4 °C for 4 min, to pellet the particulate matter. The supernatants were collected and filtered through a 0.45 μm filter, and 1 μL of sample was injected into the column. Quantitation of fecal SCFAs (acetic acid, propionic acid, butyric acid, iso-butyrate acid, valerate acid and iso-valerate acid) was performed on a GC-2014 Ultra (Shimadzu Co., Tokyo, Shimadzu) fitted with an DB-FFAP column (30 m × 0.25 μm × 0.25 μm). The column was first kept at 60 °C, and after 1 min, the column temperature escalated to 100 °C at a rate of 25 °C/min, and then to 190 °C at 15 °C/min and maintain 30 s, at last to 205 °C at 12 °C/min and maintain 60 s. Helium was used as the carrier gas at a flow rate of 0.8 mL/min and the split ratio was 50:1. Temperatures of the injector interface and ion source were 240, 250 and 220 °C, respectively.

The external standard method was used to obtain the contents of target acids in samples. SCFAs mixtures consisting of the six acids were prepared at a series of concentrations of 0, 10, 30, 50, 70, 100 and 300 μg/mL to construct the calibration curve. The calibration curve samples were treated in a similar way as the study samples.

### Statistical analysis

The correlations between the shifts in bacterial species and the changes in KEGG pathways and CAZymes, and between differential bacterial species and metabolite features (including SCFAs) were tested by Spearman rank correlation. Story’s FDR was used to correct the multiple tests. Partial least squares-discriminant analysis (PLS-DA) was performed to evaluate serum metabolite profiles of the untargeted metabolome between stunted pigs and full-sib counterparts of normal pigs (Ye et al., 2018). The Wilcoxon rank sum test was used to compare serum levels of taurine and the fecal concentrations of SCFAs between stunted and normal growth pigs at an FDR <0.05. All results were visualized by ggplot2 (v. 4.0.0).

## Data Availability

The datasets presented in this study can be found in online repositories. The names of the repository/repositories and accession number(s) can be found in the article/Supplementary material.

## Supplementary material

SUPPLEMENTARY FIGURE 1 Comparison of the ɑ-diversity of gut microbiota between stunted pigs and full-sib normal pigs in the discovery cohort as determined by 16S rRNA gene sequencing. **(A)** Observed species. **(B)** Chao. **(C)** Ace. **(D)** Simpson diversity. **(E)** Shannon index.

SUPPLEMENTARY FIGURE 2 Comparison of the ɑ-diversity of gut microbiota between stunted pigs and full-sib normal pigs in the validation cohort as determined by 16S rRNA gene sequencing. **(A)** Observed species. **(B)** Chao. **(C)** Ace. **(D)** Simpson diversity. **(E)** Shannon index.

SUPPLEMENTARY FIGURE 3 The β-diversity of gut microbiota with metagenomic sequencing data in the discovery and validation cohorts. **(A)** Discovery cohort. **(B)** Validation cohort. The β-diversity of gut mcirobiota was analyzed based on the Bray–Curtis distance. Blue dots represent stunted piglets, and red dots indicate normal growth piglets.

SUPPLEMENTARY FIGURE 4 Identification of bacterial species associated with growth retardation of pigs combining all 32 metagenomic sequencing data from discovery and validation cohorts. LEfSe analysis was used to identify differential bacterial species. LDA score ≥2 was set as the threshold.

SUPPLEMENTARY FIGURE 5 Bacterial species that could discriminate stunted pigs and normal growing pigs by random forest model in the discovery cohort **(A)** and validation cohort **(B)** separately. **(C)** Receiver operating curve (ROC) in the discovery cohort. The AUC was 92% with the 95% CI of 73.61–100%. **(D)** Receiver operating curve (ROC) in the validation cohort. The AUC was 93.06% with the 95% CI of 81.42–100%.

SUPPLEMENTARY FIGURE 6 KEGG pathways and CAZymes showing significant shifts in abundances between stunted pigs and normal growing pigs, and their relationships with differential bacterial species in the validation cohort. **(A)** Differential KEGG pathways. **(B)** Differential CAZymes. **(C,D)** The heat maps showing the relationships between differential bacterial species, and differential KEGG pathways and CAZymes. The *X*-axis represents the bacterial species. The *Y*-axis indicates the differential KEGG pathways/CAZymes. ^*^*p* < 0.05, ^**^*p* < 0.01, and ^***^*p* < 0.005.

SUPPLEMENTARY FIGURE 7 The changes in serum metabolome between stunted pigs and normal growing pigs in the validation cohort. **(A)** sPLS-DA plot of serum metabolite profiles indicating the significant differentiation of serum metabolite profiles between stunted pigs and full-sib normal pigs in the validation cohort. **(B)** The relationships of altered serum metabolite features with differential bacterial species in the discovery cohort. The *X*-axis represents bacterial species. The *Y*-axis represents the differential serum metabolite features. ^*^*p* < 0.05, ^**^*p* < 0.01, and ^***^*p* < 0.005.
