# Supplementary figures and images for: Gut microbiota regulates growth retardation in pigs through their metabolites of taurine and butyric acids
Source: Front Microbiol. 2026 Apr 17;17:1811659. doi: 10.3389/fmicb.2026.1811659 (PMC13132826; doi:10.3389/fmicb.2026.1811659)

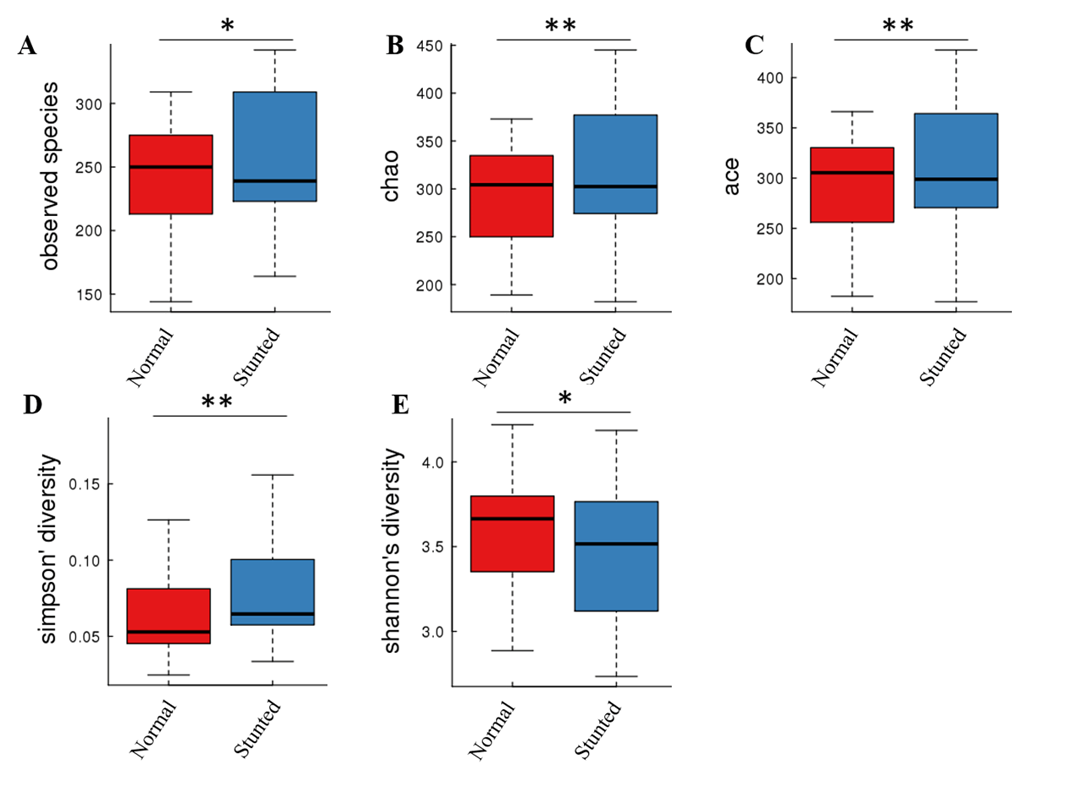

Supplement: SUPPLEMENTARY FIGURE 1 — Comparison of the ɑ-diversity of gut microbiota between stunted pigs and full-sib normal pigs in the discovery cohort as determined by 16S rRNA gene sequencing. (A) Observed species. (B) Chao. (C) Ace. (D) Simpson diversity. (E) Shannon index. [file Image_1.TIF]

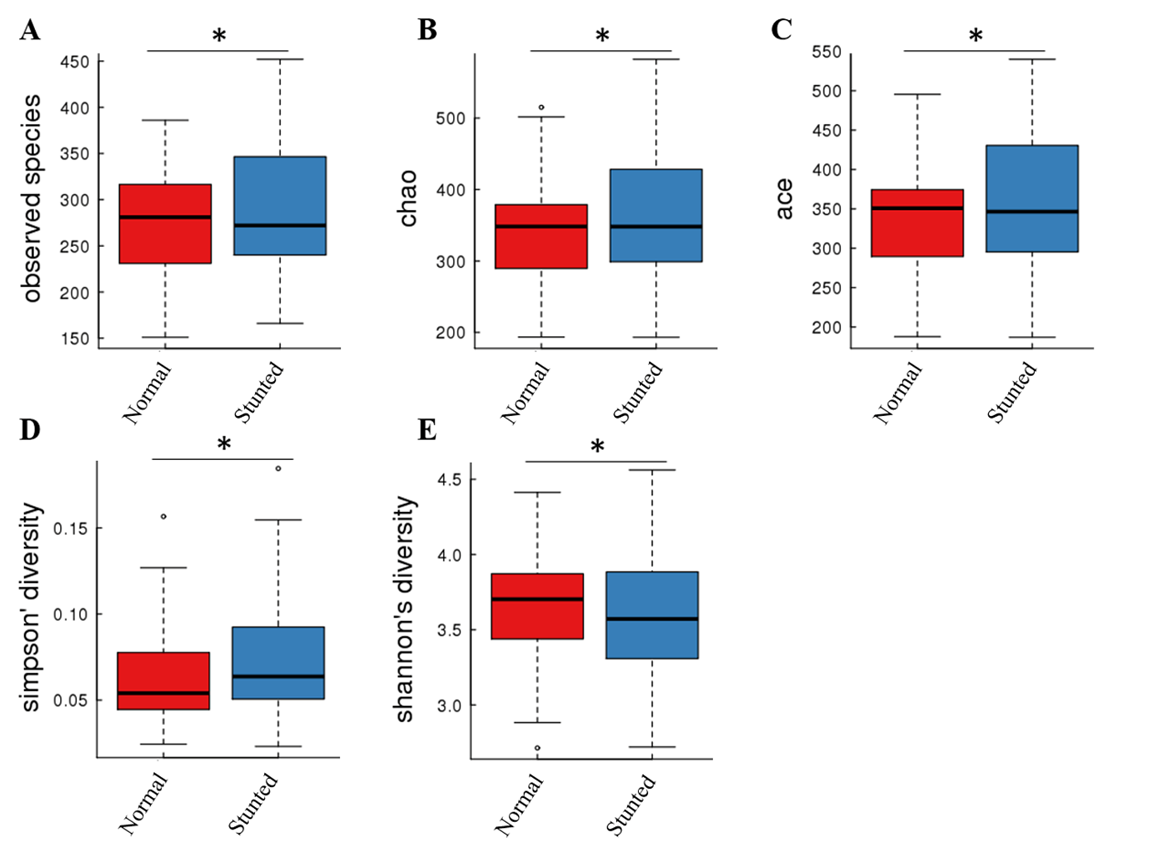

Supplement: SUPPLEMENTARY FIGURE 2 — Comparison of the ɑ-diversity of gut microbiota between stunted pigs and full-sib normal pigs in the validation cohort as determined by 16S rRNA gene sequencing. (A) Observed species. (B) Chao. (C) Ace. (D) Simpson diversity. (E) Shannon index. [file Image_2.TIF]

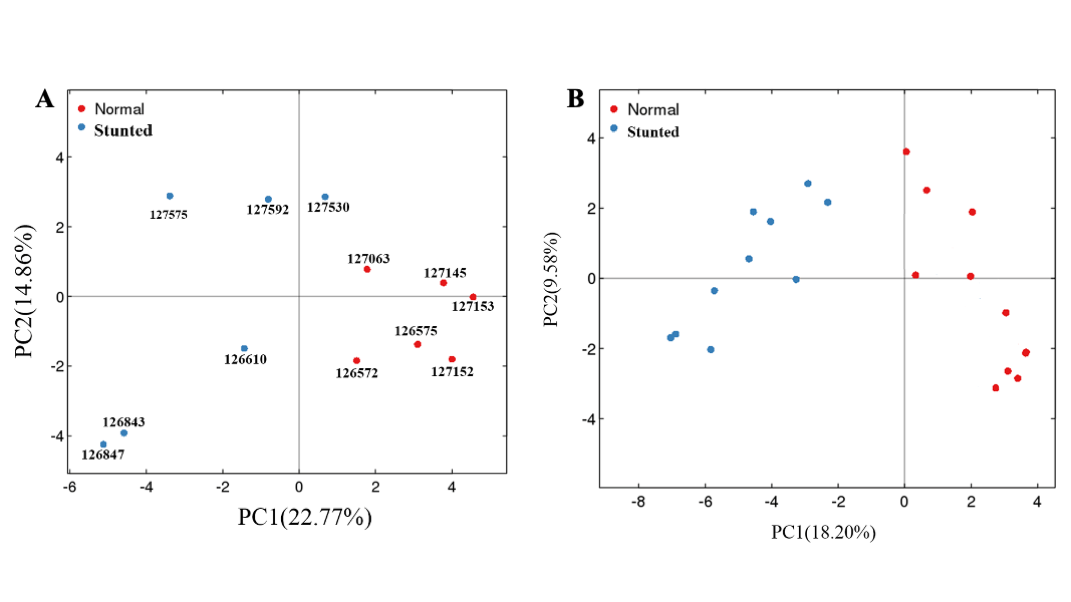

Supplement: SUPPLEMENTARY FIGURE 3 — The β-diversity of gut microbiota with metagenomic sequencing data in the discovery and validation cohorts. (A) Discovery cohort. (B) Validation cohort. The β-diversity of gut mcirobiota was analyzed based on the Bray–Curtis distance. Blue dots represent stunted piglets, and red dots indicate normal growth piglets. [file Image_3.TIFF]

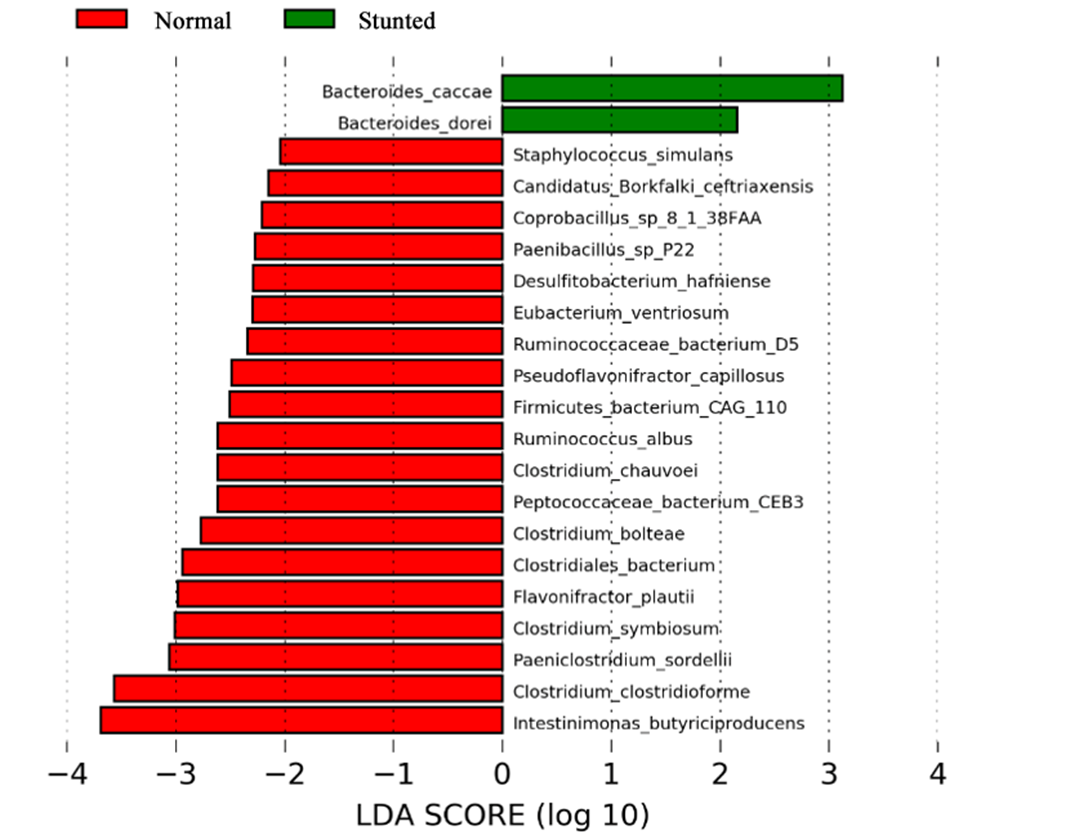

Supplement: SUPPLEMENTARY FIGURE 4 — Identification of bacterial species associated with growth retardation of pigs combining all 32 metagenomic sequencing data from discovery and validation cohorts. LEfSe analysis was used to identify differential bacterial species. LDA score ≥2 was set as the threshold. [file Image_4.TIF]

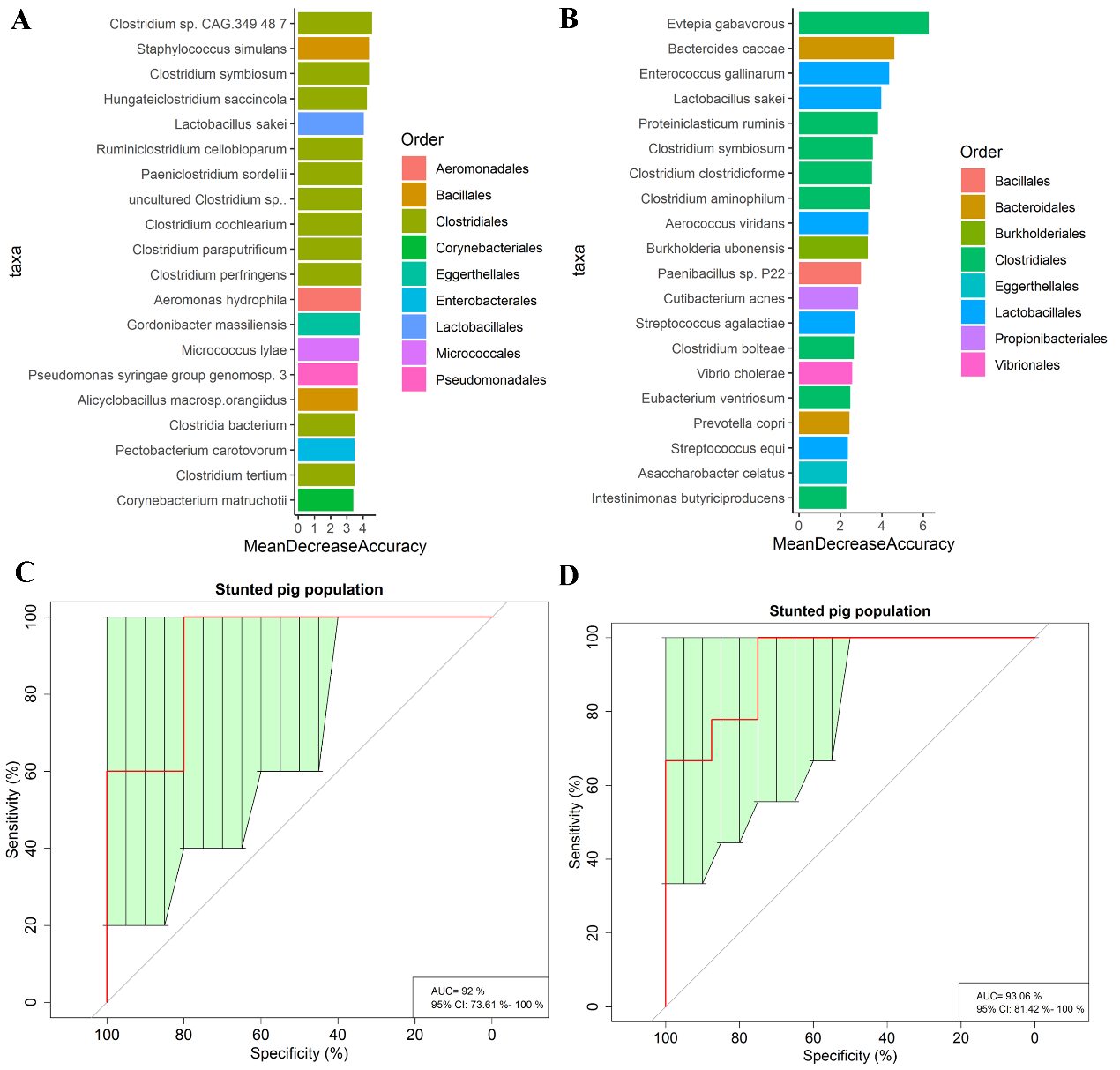

Supplement: SUPPLEMENTARY FIGURE 5 — Bacterial species that could discriminate stunted pigs and normal growing pigs by random forest model in the discovery cohort (A) and validation cohort (B) separately. (C) Receiver operating curve (ROC) in the discovery cohort. The AUC was 92% with the 95% CI of 73.61–100%. (D) Receiver operating curve (ROC) in the validation cohort. The AUC was 93.06% with the 95% CI of 81.42–100%. [file Image_5.TIF]

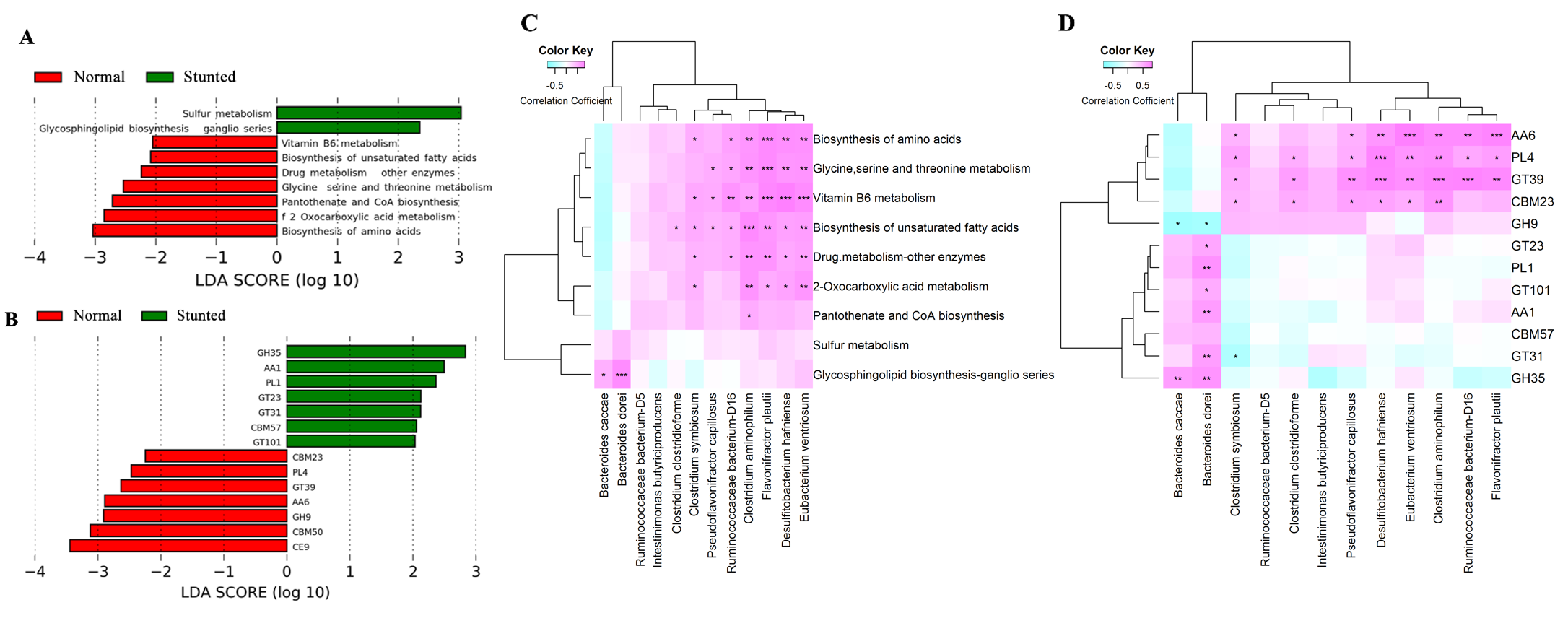

Supplement: SUPPLEMENTARY FIGURE 6 — KEGG pathways and CAZymes showing significant shifts in abundances between stunted pigs and normal growing pigs, and their relationships with differential bacterial species in the validation cohort. (A) Differential KEGG pathways. (B) Differential CAZymes. (C,D) The heat maps showing the relationships between differential bacterial species, and differential KEGG pathways and CAZymes. The X-axis represents the bacterial species. The Y-axis indicates the differential KEGG pathways/CAZymes. *p < 0.05, **p < 0.01, and ***p < 0.005. [file Image_6.TIF]

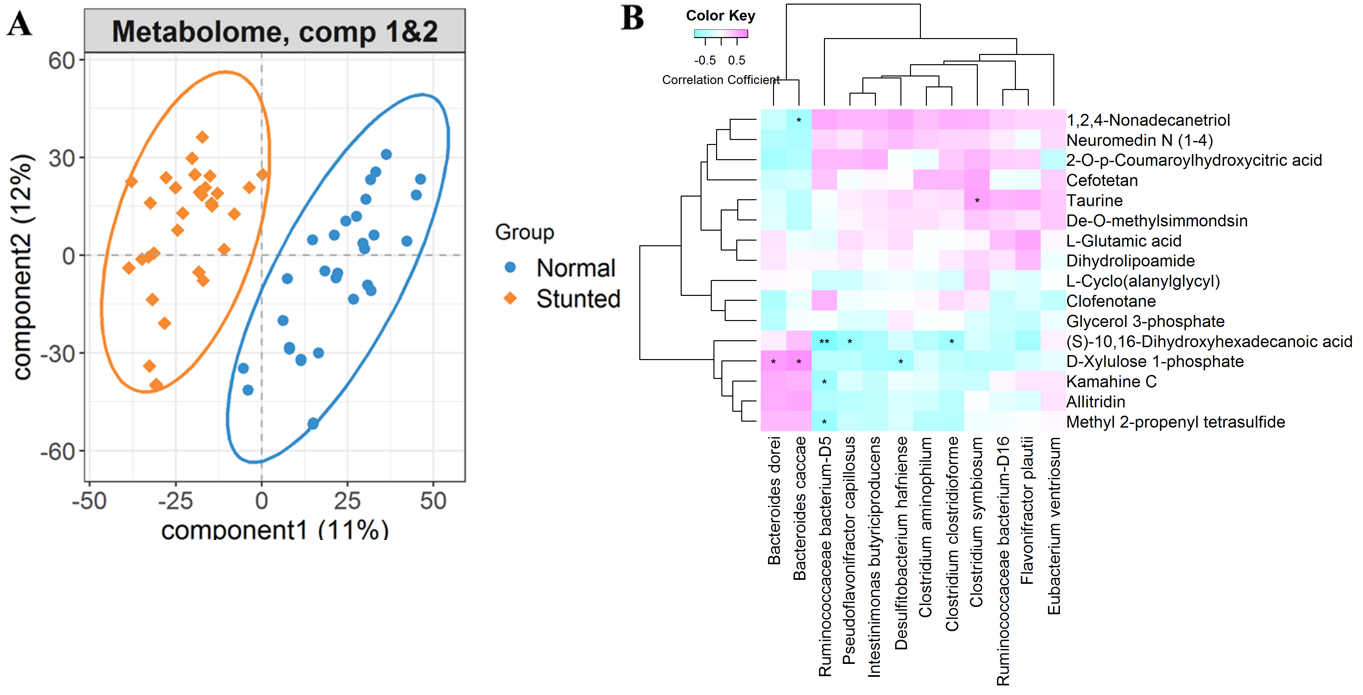

Supplement: SUPPLEMENTARY FIGURE 7 — The changes in serum metabolome between stunted pigs and normal growing pigs in the validation cohort. (A) sPLS-DA plot of serum metabolite profiles indicating the significant differentiation of serum metabolite profiles between stunted pigs and full-sib normal pigs in the validation cohort. (B) The relationships of altered serum metabolite features with differential bacterial species in the discovery cohort. The X-axis represents bacterial species. The Y-axis represents the differential serum metabolite features. *p < 0.05, **p < 0.01, and ***p < 0.005. [file Image_7.TIF]
