# Supplementary material for: Gut microbiota regulates growth retardation in pigs through their metabolites of taurine and butyric acids
Source: Front Microbiol. 2026 Apr 17;17:1811659. doi: 10.3389/fmicb.2026.1811659 (PMC13132826; doi:10.3389/fmicb.2026.1811659)
Supplement: Supplementary file 8 [file Table_1.DOCX]

**Supplementary Tables**

**Gut microbiota regulates growth retardation in pigs through their metabolites of taurine and butyric acids**

*Tao Shen^1^, Yunyan Zhou^1^, Jun Gao^1^, Xinwei Xiong^1^*, Congying Chen^1^**

*^1^ National Key Laboratory of Swine Genetic Improvement and Germplasm Innovation, Jiangxi Agricultural University, Nanchang 330045, China.*

*Corresponding author: Congying Chen and Xinwei Xiong

*National Key Laboratory of Swine Genetic Improvement and Germplasm Innovation*, Jiangxi Agricultural University, Nanchang 330045, China

Congying Chen: [chencongying@jxau.edu.cn](mailto:chencongying@jxau.edu.cn),

Xinwei Xiong: [XinweiXiong@hotmail.com](mailto:XinweiXiong@hotmail.com)

**Supplementary Table 1. OTUs showing significantly differential abundances between stunted pigs and full-sib normal growing pigs in the discovery cohort**

| **OTU level** | **Group** | **LDA value** | **P value** |
| --- | --- | --- | --- |
| Otu4_Clostridium | Normal | 4.39 | 2.04E-02 |
| Otu1_S24-7 | Normal | 4.18 | 5.69E-03 |
| Otu11_Clostridiales | Normal | 4.06 | 7.67E-03 |
| Otu98_Blautia | Normal | 3.93 | 8.87E-03 |
| Otu49_Oscillospira | Normal | 3.75 | 3.05E-03 |
| Otu66_Bacteroides | Normal | 3.57 | 4.12E-02 |
| Otu103_p.2534-18B5 | Normal | 3.54 | 4.65E-02 |
| Otu46_Clostridium | Normal | 3.49 | 1.75E-02 |
| Otu42_Butyricimonas | Normal | 3.48 | 4.91E-03 |
| Otu76_Christensenellaceae | Normal | 3.47 | 7.60E-03 |
| Otu1119_Oscillospira | Normal | 3.39 | 4.91E-02 |
| Otu1238_Christensenellaceae | Normal | 3.29 | 3.61E-03 |
| Otu223_Ruminococcaceae | Normal | 3.28 | 3.66E-02 |
| Otu17_Clostridiales | Normal | 3.22 | 1.53E-02 |
| Otu178_Treponema | Normal | 3.20 | 3.58E-02 |
| Otu229_Prevotella | Normal | 3.19 | 4.65E-02 |
| Otu37_Bacteroidales | Normal | 3.15 | 2.94E-02 |
| Otu805_Ruminococcaceae | Normal | 3.10 | 2.76E-02 |
| Otu804_Dehalobacterium | Normal | 3.09 | 3.61E-03 |
| Otu56_Synergistaceae | Normal | 3.08 | 4.67E-02 |
| Otu107_Ruminococcaceae | Normal | 3.05 | 2.04E-02 |
| Otu701_Firmicutes | Normal | 2.94 | 1.34E-02 |
| Otu117_Anaerotruncus | Normal | 2.91 | 1.03E-02 |
| Otu25_Clostridium hathewayi | Normal | 2.87 | 2.94E-02 |
| Otu238_Pyramidobacter piscolens | Normal | 2.85 | 3.19E-02 |
| Otu418_Oscillospira | Normal | 2.84 | 1.63E-02 |
| Otu68_Bacteroidales | Normal | 2.81 | 3.23E-02 |
| Otu28_Synergistaceae | Normal | 2.80 | 2.81E-02 |
| Otu256_Butyricimonas | Normal | 2.76 | 2.59E-02 |
| Otu1239_Clostridiales | Normal | 2.75 | 1.69E-03 |
| Otu388_Christensenellaceae | Normal | 2.74 | 7.71E-03 |
| Otu376_Sphaerochaeta | Normal | 2.63 | 2.45E-02 |
| Otu678_Clostridiales | Normal | 2.57 | 2.15E-02 |
| Otu12_Prevotella | Stunted | 3.76 | 2.73E-02 |
| Otu34_Lactobacillus | Stunted | 3.42 | 3.76E-02 |
| Otu130_Blautia | Stunted | 3.18 | 4.60E-02 |
| Otu321_Erysipelotrichaceae | Stunted | 2.87 | 4.49E-02 |
| Otu339_Rikenellaceae | Stunted | 2.81 | 3.13E-02 |
| Otu685_Lactobacillus salivarius | Stunted | 2.77 | 2.44E-02 |

**Supplementary Table 2. OTUs showing significantly differential abundances between stunted pigs and full-sib normal growing pigs in the validation cohort**

| OTU level | Group | LDA value | P value |
| --- | --- | --- | --- |
| Otu4_Clostridium | Normal | 4.32 | 1.83E-02 |
| Otu18_Phascolarctobacterium | Normal | 3.97 | 4.48E-02 |
| Otu7_Bacteroides | Normal | 3.75 | 1.38E-02 |
| Otu17_Clostridiales | Normal | 3.74 | 4.29E-02 |
| Otu102_Clostridium citroniae | Normal | 3.72 | 2.32E-05 |
| Otu48_Bacteroides uniformis | Normal | 3.57 | 2.57E-02 |
| Otu109_Bacteroidales | Normal | 3.40 | 4.31E-02 |
| Otu110_Lactobacillus reuteri | Normal | 3.38 | 3.97E-02 |
| Otu949_Lachnospiraceae | Normal | 3.35 | 1.67E-02 |
| Otu126_Parabacteroides | Normal | 3.32 | 1.20E-02 |
| Otu1051_Anaerotruncus | Normal | 3.06 | 6.74E-03 |
| Otu90_Mogibacteriaceae | Normal | 3.04 | 4.58E-03 |
| Otu1086_Mogibacteriaceae | Normal | 2.89 | 9.83E-03 |
| Otu1239_Clostridiales | Normal | 2.87 | 2.25E-02 |
| Otu277_Oscillospira | Normal | 2.77 | 1.27E-03 |
| Otu1157_Bacteroides | Normal | 2.76 | 6.37E-03 |
| Otu1283_Desulfovibrionaceae | Normal | 2.68 | 5.00E-02 |
| Otu1274_Ruminococcaceae | Normal | 2.60 | 1.36E-03 |
| Otu183_Oscillospira | Normal | 2.57 | 2.72E-02 |
| Otu503_Clostridiales | Normal | 2.57 | 1.82E-02 |
| Otu981_Ruminococcaceae | Normal | 2.56 | 1.32E-02 |
| Otu823_Butyricicoccus pullicaecorum | Normal | 2.55 | 1.17E-02 |
| Otu300_Ruminococcaceae | Normal | 2.54 | 4.99E-03 |
| Otu652_Anaerotruncus | Normal | 2.53 | 7.29E-03 |
| Otu931_Bacteroides | Normal | 2.51 | 1.05E-02 |
| Otu714_Mogibacteriaceae | Normal | 2.47 | 1.13E-02 |
| Otu708_Ruminococcaceae | Normal | 2.45 | 1.81E-02 |
| Otu645_Clostridiales | Normal | 2.44 | 1.32E-02 |
| Otu494_Coriobacteriaceae | Normal | 2.39 | 1.13E-02 |
| Otu549_Coriobacteriaceae | Normal | 2.24 | 4.19E-02 |
| Otu65_Prevotella | Stunted | 3.54 | 1.86E-02 |
| Otu1115_Oscillospira | Stunted | 3.38 | 4.27E-03 |
| Otu51_Campylobacter | Stunted | 3.37 | 5.81E-03 |
| Otu137_Christensenellaceae | Stunted | 3.31 | 2.47E-02 |
| Otu24_Christensenellaceae | Stunted | 3.24 | 4.70E-03 |
| Otu23_Prevotella | Stunted | 3.19 | 3.84E-02 |
| Otu114_CF231 | Stunted | 3.10 | 1.43E-02 |
| Otu297_S24-7 | Stunted | 3.07 | 1.02E-02 |
| Otu195_Prevotella | Stunted | 3.07 | 1.09E-02 |
| Otu173_Anaerovibrio | Stunted | 3.03 | 2.11E-02 |
| Otu278_Lachnospiraceae | Stunted | 3.03 | 2.99E-02 |
| Otu69_Prevotella | Stunted | 2.82 | 3.09E-02 |
| Otu259_S24-7 | Stunted | 2.74 | 3.71E-02 |
| Otu125_Clostridiales | Stunted | 2.73 | 2.97E-02 |
| Otu305_Prevotella | Stunted | 2.67 | 2.99E-02 |
| Otu607_Desulfovibrionaceae | Stunted | 2.65 | 4.66E-03 |
| Otu111_Faecalibacterium_prausnitzii | Stunted | 2.62 | 1.81E-02 |
| Otu261_Ruminococcaceae | Stunted | 2.58 | 3.58E-05 |
| Otu224_Ruminococcaceae | Stunted | 2.55 | 3.72E-03 |
| Otu263_Prevotella_stercorea | Stunted | 2.55 | 3.79E-02 |
| Otu548_Ruminococcaceae | Stunted | 2.53 | 3.95E-02 |
| Otu316_Oscillospira | Stunted | 2.52 | 1.02E-02 |
| Otu35_Prevotella | Stunted | 2.51 | 2.99E-02 |
| Otu640_Oscillospira | Stunted | 2.51 | 4.05E-02 |
| Otu770_Lachnospiraceae | Stunted | 2.51 | 9.25E-03 |
| Otu377_RFN20 | Stunted | 2.50 | 3.58E-02 |
| Otu900_Ruminococcaceae | Stunted | 2.48 | 4.07E-02 |
| Otu677_Oscillospira | Stunted | 2.43 | 3.28E-02 |
| Otu253_S24-7 | Stunted | 2.41 | 1.02E-02 |
| Otu1080_Sphaerochaeta | Stunted | 2.38 | 2.54E-02 |
| Otu141_Lachnospiraceae | Stunted | 2.31 | 2.28E-02 |
| Otu64_Sphaerochaeta | Stunted | 2.29 | 3.41E-02 |
| Otu1074_Oscillospira | Stunted | 2.15 | 3.57E-02 |

**Supplementary Table 3. Summary description of metagenomic sequencing data**

| **Sample ID** | **Group** | **Total length (bp)** | **Read No.** | **Average length (bp)** | **Max length (bp)** | **N50 (bp)** | **N90 (bp)** |
| --- | --- | --- | --- | --- | --- | --- | --- |
| sample01 | Stunted | 245,646,717 | 125,821 | 1,952.35 | 310,940 | 4,210 | 670 |
| sample02 | Stunted | 648,803,727 | 833,138 | 778.75 | 322,682 | 746 | 536 |
| sample03 | Stunted | 301,652,378 | 191,397 | 1,576.06 | 437,654 | 2,170 | 626 |
| sample04 | Stunted | 239,910,236 | 116,367 | 2,061.67 | 308,611 | 4,390 | 705 |
| sample05 | Stunted | 359,841,941 | 363,085 | 991.07 | 261,509 | 863 | 536 |
| sample06 | Stunted | 876,305,448 | 1,086,032 | 806.89 | 234,548 | 800 | 546 |
| sample07 | Normal | 291,761,609 | 148,095 | 1,970.10 | 286,734 | 4,142 | 682 |
| sample08 | Normal | 264,156,618 | 137,189 | 1,925.49 | 311,606 | 3,370 | 689 |
| sample09 | Normal | 253,358,141 | 117,510 | 2,156.06 | 761,807 | 4,826 | 713 |
| sample10 | Normal | 263,634,987 | 132,116 | 1,995.48 | 722,641 | 3,993 | 694 |
| sample11 | Normal | 256,646,172 | 124,006 | 2,069.63 | 382,918 | 4,712 | 693 |
| sample12 | Normal | 260,171,306 | 133,047 | 1,955.48 | 340,330 | 3,746 | 686 |
| sample13 | Stunted | 161,927,246 | 64,722 | 2,501.89 | 408,228 | 8,894 | 749 |
| sample14 | Stunted | 189,822,967 | 76,701 | 2,474.84 | 387,933 | 6,633 | 786 |
| sample15 | Stunted | 170,141,967 | 87,562 | 1,943.10 | 589,748 | 3,860 | 673 |
| sample16 | Stunted | 104,340,211 | 85,678 | 1,217.82 | 390,237 | 1,492 | 545 |
| sample17 | Stunted | 151,335,274 | 87,000 | 1,739.49 | 368,725 | 2,776 | 657 |
| sample18 | Stunted | 160,867,552 | 97,796 | 1,644.93 | 492,511 | 2,512 | 635 |
| sample19 | Stunted | 224,251,660 | 118,644 | 1,890.12 | 438,955 | 3,371 | 678 |
| sample20 | Stunted | 121,248,386 | 99,344 | 1,220.49 | 370,729 | 1,475 | 544 |
| sample21 | Stunted | 129,281,995 | 69,003 | 1,873.57 | 476,626 | 4,107 | 630 |
| sample22 | Stunted | 170,573,368 | 90,621 | 1,882.27 | 438,868 | 3,425 | 676 |
| sample23 | Normal | 195,592,579 | 83,442 | 2,344.05 | 254,666 | 5,024 | 774 |
| sample24 | Normal | 197,707,695 | 89,314 | 2,213.62 | 592,496 | 5,439 | 713 |
| sample25 | Normal | 154,430,111 | 79,172 | 1,950.56 | 465,557 | 3,690 | 685 |
| sample26 | Normal | 196,009,341 | 262,157 | 747.68 | 238,614 | 666 | 522 |
| sample27 | Normal | 159,953,379 | 63,541 | 2,517.33 | 558,341 | 7,138 | 790 |
| sample28 | Normal | 193,422,424 | 87,987 | 2,198.31 | 768,060 | 4,782 | 733 |
| sample29 | Normal | 172,716,624 | 69,192 | 2,496.19 | 393,425 | 8,609 | 760 |
| sample30 | Normal | 197,818,435 | 101,366 | 1,951.53 | 457,626 | 3,483 | 698 |
| sample31 | Normal | 106,318,122 | 48,047 | 2,212.79 | 380,442 | 5,458 | 722 |
| sample32 | Normal | 184,559,247 | 99,310 | 1,858.42 | 452,237 | 3,321 | 663 |

**Supplementary Table 4. Metabolite features showing differential abundances between stunted pigs and full-sib normal growing pigs in the discovery cohort**

| **Retention time (RT)–m/z** | **Group** | **P value** | **FDR** | **Putative compound** |
| --- | --- | --- | --- | --- |
| 0.78_124.0765m/z | Stunted | 1.60E-05 | 2.82E-03 | 1-Ethyl-1H-pyrrole-2-carboxaldehyde |
| 9.51_261.8515n | Normal | 1.15E-05 | 2.82E-03 | 1-Bromo-3-iodoacetone |
| 9.13_322.7974m/z | Normal | 8.11E-06 | 2.82E-03 | Aluminium sulfate |
| 0.80_131.0462m/z | Stunted | 3.95E-06 | 2.82E-03 | D-Asparagine |
| 12.19_339.3259m/z | Normal | 1.60E-05 | 2.82E-03 | 6-Hydroxy-8-docosanone |
| 0.83_131.0826m/z | Stunted | 8.11E-06 | 2.82E-03 | L-Ornithine monochlorohydrate/ornithine |
| 0.83_147.0531n | Stunted | 1.60E-05 | 2.82E-03 | L-Glutamic acid |
| 0.75_335.0447m/z | Stunted | 1.48E-05 | 2.82E-03 | 2-O-p-Coumaroylhydroxycitric acid |
| 0.83_102.0561m/z | Stunted | 1.89E-05 | 2.95E-03 | Gamma-Aminobutyric acid |
| 14.44_334.1472m/z | Stunted | 2.22E-05 | 3.12E-03 | Hydroxytyrosol 1-O-glucoside |
| 12.76_251.0344m/z | Normal | 3.05E-05 | 3.13E-03 | Taurine |
| 0.77_112.9856m/z | Stunted | 2.60E-05 | 3.13E-03 | trifluoroacetic acid |
| 0.77_248.9606m/z | Stunted | 3.56E-05 | 3.13E-03 | 5-(4-Chloro-3-hydroxy-1-butynyl)-2,2'-bithiophene |
| 0.77_827.3548m/z | Stunted | 2.79E-05 | 3.13E-03 | Neopetasitenine |
| 0.77_316.9481m/z | Stunted | 3.56E-05 | 3.13E-03 | Mitotane |
| 0.81_132.0304m/z | Stunted | 3.30E-05 | 3.13E-03 | 2-Hydroxyadenine |
| 12.16_291.1249m/z | Stunted | 4.83E-05 | 3.50E-03 | 5,6-Dihydro-11-methoxyyangonin |
| 1.21_307.0346m/z | Stunted | 4.67E-05 | 3.50E-03 | dUMP |
| 0.84_217.0302m/z | Stunted | 4.83E-05 | 3.50E-03 | Cysteinyl-Aspartate |
| 0.77_1039.3060m/z | Stunted | 4.98E-05 | 3.50E-03 | Gadobenate Dimeglumine |
| 11.63_212.0025m/z | Stunted | 6.51E-05 | 4.36E-03 | Indoxyl sulfate |
| 11.80_325.3104m/z | Normal | 8.69E-05 | 5.29E-03 | Heneicosanoic acid |
| 0.77_759.3673m/z | Stunted | 9.03E-05 | 5.29E-03 | Vinorelbine |
| 1.20_263.0080m/z | Stunted | 8.86E-05 | 5.29E-03 | 5,8-Dihydro-6-(4-methyl-3-pentenyl)-1,2,3,4-tetrathiocin |
| 3.44_296.9763m/z | Stunted | 1.00E-04 | 5.64E-03 | Dithianon |
| 11.35_210.9989m/z | Stunted | 1.15E-04 | 6.01E-03 | D-Xylulose 1-phosphate |
| 9.64_495.1492m/z | Stunted | 1.15E-04 | 6.01E-03 | Musabalbisiane A |
| 0.84_215.0327m/z | Stunted | 1.25E-04 | 6.28E-03 | 7-Acetoxy-6-methoxycoumarin |
| 0.83_152.9956m/z | Stunted | 1.32E-04 | 6.43E-03 | Glycerol 3-phosphate |
| 15.99_703.5790m/z | Stunted | 1.52E-04 | 6.90E-03 | Glycerol tritridecanoate |
| 0.83_168.0426m/z | Stunted | 1.52E-04 | 6.90E-03 | 6-Chloro-N-(1-methylethyl)-1,3,5-triazine-2,4-diamine |
| 5.81_207.1389m/z | Stunted | 1.57E-04 | 6.91E-03 | 4,5-Dihydrovomifoliol |
| 14.53_362.1460m/z | Stunted | 1.99E-04 | 8.23E-03 | De-O-methylsimmondsin |
| 10.16_235.8437m/z | Normal | 1.99E-04 | 8.23E-03 | Amino (methoxysulfinyl) pentasulfide |
| 0.75_213.0856m/z | Stunted | 2.19E-04 | 8.82E-03 | Aspartyl-Valine |
| 5.29_236.1263m/z | Stunted | 2.27E-04 | 8.87E-03 | Pandamarilactam 3x |
| 4.56_182.9540m/z | Normal | 2.59E-04 | 9.83E-03 | Mercury |
| 0.82_242.0799m/z | Stunted | 2.74E-04 | 9.88E-03 | Gamma-glutamyl-Aspartate |
| 0.83_294.0690m/z | Stunted | 2.72E-04 | 9.88E-03 | (S)-5'-Deoxy-5'-(methylsulfinyl)adenosine |
| 4.86_245.0240m/z | Stunted | 2.94E-04 | 1.01E-02 | S-(2,5-Dimethyl-3-furanyl) 2-furancarbothioate |
| 11.01_269.2482m/z | Normal | 2.94E-04 | 1.01E-02 | (卤)-11-Methylhexadecanoic acid |
| 13.87_365.1505m/z | Stunted | 3.34E-04 | 1.09E-02 | 1,2-Dimethoxy-13-methyl-[1,3]benzodioxolo[5,6-c]phenanthridine |
| 9.65_419.1367m/z | Stunted | 3.34E-04 | 1.09E-02 | Cnidimol 7-glucoside |
| 13.37_249.1159m/z | Normal | 3.79E-04 | 1.21E-02 | Kamahine C |
| 14.13_221.1301m/z | Stunted | 4.29E-04 | 1.27E-02 | Methsuximide |
| 5.03_236.1579m/z | Stunted | 4.29E-04 | 1.27E-02 | Pantothenamide |
| 0.75_145.0982m/z | Stunted | 4.29E-04 | 1.27E-02 | (3S,5S)-3,5-Diaminohexanoate |
| 0.80_104.0353m/z | Stunted | 4.32E-04 | 1.27E-02 | L-Serine |
| 1.21_158.9754m/z | Stunted | 4.85E-04 | 1.37E-02 | Allitridin |
| 9.66_579.0821m/z | Stunted | 4.85E-04 | 1.37E-02 | Epitheaflavic acid 3'-gallate |
| 12.07_458.1644m/z | Normal | 5.48E-04 | 1.43E-02 | Mandelonitrile sophoroside |
| 1.89_191.9400m/z | Stunted | 5.48E-04 | 1.43E-02 | Bis(methylthio) selenide |
| 12.03_297.2797m/z | Normal | 5.48E-04 | 1.43E-02 | 1,2,4-Nonadecanetriol |
| 0.81_333.0591m/z | Stunted | 5.19E-04 | 1.43E-02 | Chlorogenoquinone |
| 0.81_203.0203m/z | Stunted | 5.74E-04 | 1.47E-02 | Daucic acid |
| 0.81_146.0691n | Stunted | 6.18E-04 | 1.55E-02 | Alanyl-Glycine |
| 3.74_249.1205m/z | Stunted | 6.96E-04 | 1.68E-02 | 6-Hydroxymelatonin |
| 12.62_221.0806m/z | Normal | 6.96E-04 | 1.68E-02 | Ethyl 3,4,5-trimethoxybenzoate |
| 0.81_109.0407m/z | Stunted | 7.05E-04 | 1.68E-02 | L-Cyclo(alanylglycyl) |
| 10.43_340.1695m/z | Stunted | 7.83E-04 | 1.84E-02 | N1-(2-Methoxy-4-methylbenzyl)-n2-(2-(5-methylpyridin-2-yl)ethyl)oxalamide |
| 3.10_406.0250m/z | Stunted | 8.68E-04 | 1.99E-02 | Thiamine pyrophosphate |
| 11.46_316.9206m/z | Normal | 8.79E-04 | 1.99E-02 | Tiludronate |
| 15.83_169.1594m/z | Stunted | 9.86E-04 | 2.17E-02 | 10-Undecenal |
| 8.49_327.1902m/z | Stunted | 9.86E-04 | 2.17E-02 | Metixene |
| 0.81_411.9688m/z | Stunted | 1.04E-03 | 2.25E-02 | Benzthiazide |
| 6.35_229.1822m/z | Stunted | 1.10E-03 | 2.32E-02 | 11-Hydroxy-9-tridecenoic acid |
| 11.64_485.2781m/z | Normal | 1.10E-03 | 2.32E-02 | Neuromedin N (1-4) |
| 10.80_327.1880m/z | Normal | 1.23E-03 | 2.52E-02 | Metixene |
| 11.47_465.1020m/z | Normal | 1.23E-03 | 2.52E-02 | Diospyrin |
| 0.82_166.0177m/z | Stunted | 1.29E-03 | 2.53E-02 | Homocysteinesulfinic acid |
| 9.64_453.0881m/z | Stunted | 1.27E-03 | 2.53E-02 | (-)-Epigallocatechin 3-(4-methyl-gallate) |
| 0.80_297.9679m/z | Stunted | 1.29E-03 | 2.53E-02 | [Nitrilotris(methylene)]trisphosphonic acid |
| 14.77_230.0625m/z | Stunted | 1.38E-03 | 2.59E-02 | Dihydrolipoamide |
| 13.86_371.2160m/z | Stunted | 1.38E-03 | 2.59E-02 | [8]-Paradyl acetate |
| 11.52_555.9834m/z | Normal | 1.38E-03 | 2.59E-02 | Cefotetan |
| 0.76_336.9201m/z | Stunted | 1.70E-03 | 3.14E-02 | Selenocystine |
| 3.98_248.0279m/z | Stunted | 1.91E-03 | 3.40E-02 | Diazoxide |
| 12.08_152.9866m/z | Normal | 1.91E-03 | 3.40E-02 | 2,4,6-Trithiaheptane |
| 1.23_332.8990m/z | Stunted | 1.91E-03 | 3.40E-02 | Clofenotane |
| 4.01_354.1831n | Stunted | 2.12E-03 | 3.60E-02 | Acetylsalvipisone |
| 3.73_265.1559m/z | Stunted | 2.12E-03 | 3.60E-02 | Phenylalanyl-Valine |
| 0.81_179.9312m/z | Stunted | 2.12E-03 | 3.60E-02 | Tellurite |
| 8.57_269.2119m/z | Normal | 2.12E-03 | 3.60E-02 | (S)-10,16-Dihydroxyhexadecanoic acid |
| 0.80_280.9855m/z | Stunted | 2.35E-03 | 3.90E-02 | Acetylphosphate |
| 4.51_285.1700m/z | Stunted | 2.35E-03 | 3.90E-02 | 6,7-Dihydro-4-(hydroxymethyl)-2-(p-hydroxyphenethyl)-7-methyl-5H-2-pyrindinium |
| 4.29_333.0869m/z | Stunted | 2.61E-03 | 4.13E-02 | Fospropofol |
| 11.77_334.1413m/z | Stunted | 2.61E-03 | 4.13E-02 | Lansamide 4 |
| 8.01_170.0819m/z | Stunted | 2.61E-03 | 4.13E-02 | Pyridoxine |
| 11.38_589.1382m/z | Normal | 2.61E-03 | 4.13E-02 | Proanthocyanidin A2 |
| 0.79_445.0534m/z | Stunted | 2.74E-03 | 4.28E-02 | CDP-Ethanolamine |
| 11.68_522.1822m/z | Normal | 2.89E-03 | 4.38E-02 | 6-Caffeoylsucrose |
| 3.74_178.1077m/z | Stunted | 2.89E-03 | 4.38E-02 | N-Methyl-1-deoxynojirimycin |
| 2.87_184.9581m/z | Normal | 2.89E-03 | 4.38E-02 | Methyl 2-propenyl tetrasulfide |
| 6.32_267.1312m/z | Stunted | 3.20E-03 | 4.55E-02 | Phenylalanyl-Threonine |
| 5.29_297.0585m/z | Stunted | 3.20E-03 | 4.55E-02 | Enilconazole |
| 0.82_159.0657m/z | Stunted | 3.20E-03 | 4.55E-02 | Isopropylmaleate |
| 16.00_380.2439m/z | Stunted | 3.20E-03 | 4.55E-02 | (R)-Humulone |
| 10.91_313.1896m/z | Normal | 3.20E-03 | 4.55E-02 | Rhazidigenine Nb-oxide |
| 11.22_459.3257m/z | Normal | 3.20E-03 | 4.55E-02 | all-trans-Carophyll yellow |

**Supplementary Table 5. Metabolite features showing differential abundances between stunted pigs and full-sib normal growing pigs in the validation cohort**

| **Retention time (RT)–m/z** | **Group** | **P value** | **FDR** | **Putative compound** |
| --- | --- | --- | --- | --- |
| 8.36_275.2009m/z | Stunted | 1.52E-11 | 6.13E-08 | (9S,10E,12Z,15Z)-9-Hydroxy-10,12,15-octadecatrienoic acid |
| 8.37_388.1637n | Stunted | 1.07E-10 | 2.15E-07 | Nisoldipine |
| 9.02_425.1547m/z | Stunted | 4.14E-09 | 5.56E-06 | Furomammea D |
| 7.79_223.1698m/z | Stunted | 5.68E-09 | 5.72E-06 | L-Menthyl (R,S)-3-hydroxybutyrate |
| 7.32_267.1964m/z | Stunted | 7.74E-09 | 6.23E-06 | 16-Hydroxy-10-oxohexadecanoic acid |
| 9.64_883.6637m/z | Stunted | 1.39E-08 | 8.02E-06 | Coenzyme Q10 |
| 13.73_473.2816m/z | Normal | 1.85E-08 | 8.26E-06 | Demethylphylloquinone |
| 6.47_215.1646m/z | Stunted | 5.34E-08 | 1.79E-05 | xi-5-Hydroxydodecanoic acid |
| 7.60_243.1960m/z | Stunted | 5.34E-08 | 1.79E-05 | 2-Hydroxymyristic acid |
| 6.52_239.1649m/z | Stunted | 6.86E-08 | 1.97E-05 | Tetradecanedioic acid |
| 13.21_168.9893m/z | Normal | 8.75E-08 | 2.20E-05 | Dihydroxyacetone phosphate |
| 9.24_451.1630m/z | Stunted | 8.75E-08 | 2.20E-05 | Phenethyl rutinoside |
| 14.72_511.2737m/z | Normal | 1.11E-07 | 2.64E-05 | Ganoderic acid N |
| 8.93_345.2022m/z | Stunted | 1.41E-07 | 3.15E-05 | 1-Acetoxy-2-hydroxy-16-heptadecyn-4-one |
| 7.28_233.1543m/z | Normal | 2.77E-07 | 5.58E-05 | 2,3-Dihydroabscisic alcohol |
| 11.72_444.2551n | Normal | 3.45E-07 | 6.31E-05 | Antibiotic GR 95647X |
| 11.72_359.1539m/z | Normal | 3.45E-07 | 6.31E-05 | 15-Hydroxyleptocarpin |
| 9.64_614.4686n | Stunted | 4.27E-07 | 6.87E-05 | Donhexocin |
| 9.63_581.4430m/z | Stunted | 4.27E-07 | 6.87E-05 | Phytoene |
| 9.55_397.2372m/z | Stunted | 5.27E-07 | 8.15E-05 | Alfentanil |
| 7.61_300.1025m/z | Normal | 6.48E-07 | 9.31E-05 | Imipenem |
| 11.72_443.2383m/z | Normal | 6.48E-07 | 9.31E-05 | Apo-10'-violaxanthal |
| 10.53_425.1236m/z | Stunted | 7.93E-07 | 1.06E-04 | Citromitin |
| 17.92_427.2347m/z | Normal | 1.18E-06 | 1.40E-04 | Cinnzeylanine |
| 7.60_247.0885m/z | Normal | 1.18E-06 | 1.40E-04 | Lamivudine |
| 7.62_277.0953m/z | Normal | 1.18E-06 | 1.40E-04 | Nepafenac |
| 9.65_347.2190m/z | Stunted | 1.73E-06 | 1.74E-04 | Cortolone |
| 5.81_800.3446m/z | Normal | 1.73E-06 | 1.74E-04 | Fumonisin A1 |
| 9.64_364.2074m/z | Stunted | 1.73E-06 | 1.74E-04 | 3-Oxododecanoic acid glycerides |
| 6.24_538.0692m/z | Normal | 1.73E-06 | 1.74E-04 | Narceinone |
| 15.34_556.5288m/z | Normal | 2.09E-06 | 1.96E-04 | Armillaramide |
| 11.98_982.9759m/z | Normal | 2.09E-06 | 1.96E-04 | Diguanosine pentaphosphate |
| 7.92_205.1598m/z | Normal | 2.09E-06 | 1.96E-04 | Linalyl butyrate |
| 9.55_432.1822n | Stunted | 2.52E-06 | 2.20E-04 | (2S,4S,6S)-2-[2-(4-Hydroxy-3-meyhoxyphenyl)ethyl]tetrahydro-6-  (4,5-dihydroxy-3-methoxyphenyl)-2H-pyran-4-yl 4-acetate |
| 8.76_369.2059m/z | Stunted | 2.52E-06 | 2.20E-04 | 17a,21-Dihydroxypreg-nenolone |
| 9.32_451.1657m/z | Stunted | 2.52E-06 | 2.20E-04 | Marmesin rhamnoside |
| 17.52_568.5633m/z | Normal | 3.02E-06 | 2.59E-04 | Cer(d18:0/18:0) |
| 15.42_512.5013m/z | Normal | 3.61E-06 | 2.80E-04 | Cer(d18:0/14:0) |
| 14.68_293.1826m/z | Normal | 3.61E-06 | 2.80E-04 | Sodium Tetradecyl Sulfate |
| 9.43_382.1930n | Stunted | 3.61E-06 | 2.80E-04 | Tofisopam |
| 9.56_285.2584m/z | Stunted | 3.61E-06 | 2.80E-04 | 3-Pentadecylphenol |
| 9.56_547.2484m/z | Stunted | 3.61E-06 | 2.80E-04 | Canescein |
| 9.94_405.1700m/z | Stunted | 4.31E-06 | 3.22E-04 | beta-Mangostin |
| 9.20_511.1378m/z | Stunted | 4.31E-06 | 3.22E-04 | Emodinanthranol |
| 9.11_265.2164m/z | Stunted | 5.14E-06 | 3.69E-04 | Muricatacin |
| 6.77_488.2984m/z | Normal | 5.14E-06 | 3.69E-04 | Dynorphin A (6-8) |
| 10.54_400.1456m/z | Stunted | 6.10E-06 | 4.31E-04 | Droperidol |
| 7.60_376.1279m/z | Normal | 7.23E-06 | 4.77E-04 | Dihydrocaffeic acid 3-O-glucuronide |
| 14.56_210.8601m/z | Stunted | 7.23E-06 | 4.77E-04 | Pyrophosphate |
| 6.79_424.0569m/z | Stunted | 8.55E-06 | 5.38E-04 | Ochratoxin A |
| 9.93_305.2477m/z | Stunted | 1.01E-05 | 6.06E-04 | (Z)-15-Oxo-11-eicosenoic acid |
| 11.12_210.9992m/z | Stunted | 1.01E-05 | 6.06E-04 | D-Xylulose 1-phosphate |
| 5.88_376.2270m/z | Normal | 1.01E-05 | 6.06E-04 | trans-2-Dodecenoylcarnitine |
| 7.60_279.1136m/z | Normal | 1.19E-05 | 6.46E-04 | flumequine |
| 8.01_249.1860m/z | Stunted | 1.19E-05 | 6.46E-04 | 3-Methyl-alpha-ionyl acetate |
| 3.58_212.0922m/z | Stunted | 1.19E-05 | 6.46E-04 | Suberylglycine |
| 8.91_577.4224m/z | Stunted | 1.19E-05 | 6.46E-04 | 2-Hexaprenyl-3-methyl-5-hydroxy-6-methoxy-1,4-benzoquinol |
| 9.38_353.1354m/z | Stunted | 1.19E-05 | 6.46E-04 | Deacetyldiltiazem |
| 8.76_401.1394m/z | Stunted | 1.39E-05 | 7.02E-04 | Cyclomulberrin |
| 9.38_339.1953n | Stunted | 1.39E-05 | 7.02E-04 | Methylergonovine |
| 9.20_467.1417m/z | Stunted | 1.39E-05 | 7.02E-04 | Armillaricin |
| 9.64_380.1636n | Stunted | 1.63E-05 | 7.23E-04 | Mangostinone |
| 9.64_356.1961n | Stunted | 1.63E-05 | 7.23E-04 | Piperochromanoic acid |
| 9.56_308.2731n | Stunted | 1.63E-05 | 7.23E-04 | Sclareol |
| 6.22_478.2184n | Normal | 1.63E-05 | 7.23E-04 | 4-Hydroxyandrostenedione glucuronide |
| 2.27_192.9331m/z | Stunted | 1.63E-05 | 7.23E-04 | Ferrocyanide salts |
| 9.56_479.1833m/z | Stunted | 1.63E-05 | 7.23E-04 | Conivaptan |
| 14.30_484.4692m/z | Normal | 1.91E-05 | 7.78E-04 | Cer(d18:0/12:0) |
| 2.94_216.9792m/z | Stunted | 1.91E-05 | 7.78E-04 | 5-Sulfosalicylic acid |
| 9.64_423.1084m/z | Stunted | 1.91E-05 | 7.78E-04 | Chrysaloin |
| 8.59_671.4146m/z | Normal | 1.91E-05 | 7.78E-04 | Mactraxanthin |
| 10.53_386.2006n | Stunted | 1.91E-05 | 7.78E-04 | Sufentanil |
| 9.63_621.3916m/z | Stunted | 1.91E-05 | 7.78E-04 | 3'-N-Acetyl-4'-O-(14-methylheptadecanoyl)fusarochromanone |
| 7.37_334.1061m/z | Normal | 2.23E-05 | 7.88E-04 | Norisodomesticine |
| 7.59_152.0485m/z | Normal | 2.23E-05 | 7.88E-04 | Quinoline |
| 9.66_221.1060m/z | Normal | 2.23E-05 | 7.88E-04 | Tacrine |
| 9.98_267.2329m/z | Stunted | 2.23E-05 | 7.88E-04 | Avocadene |
| 9.37_348.1296n | Stunted | 2.23E-05 | 7.88E-04 | Torasemide |
| 13.74_391.3593m/z | Normal | 2.23E-05 | 7.88E-04 | 1,24-Tetracosanediol |
| 5.48_440.0524m/z | Stunted | 2.23E-05 | 7.88E-04 | De-O-methylsimmondsin |
| 4.22_245.0477m/z | Stunted | 2.23E-05 | 7.88E-04 | Sulfamerazine |
| 3.07_429.1555m/z | Normal | 2.23E-05 | 7.88E-04 | 8-Acetoxy-4'-methoxypinoresinol |
| 0.94_290.0871m/z | Stunted | 2.23E-05 | 7.88E-04 | N-Acetylneuraminic acid |
| 9.51_401.1531m/z | Stunted | 2.23E-05 | 7.88E-04 | tiracizine |
| 13.48_284.9624m/z | Normal | 2.60E-05 | 8.73E-04 | Homocitric acid |
| 9.19_677.4470m/z | Stunted | 2.60E-05 | 8.73E-04 | 1,2-Di-(9Z,12Z-octadecadienoyl)-sn-glycero-3-phosphate |
| 6.25_558.1224m/z | Normal | 2.60E-05 | 8.73E-04 | Gadoteridol |
| 9.13_315.2272m/z | Stunted | 2.60E-05 | 8.73E-04 | Methyl linoleate |
| 9.43_485.1416m/z | Stunted | 2.60E-05 | 8.73E-04 | PSF-A |
| 9.96_367.1617m/z | Stunted | 2.60E-05 | 8.73E-04 | Testosterone sulfate |
| 9.18_555.9811m/z | Normal | 3.02E-05 | 9.59E-04 | Cefotetan |
| 10.54_410.1751m/z | Stunted | 3.02E-05 | 9.59E-04 | Dihydroxyfumitremorgin C |
| 6.69_380.2536m/z | Normal | 3.02E-05 | 9.59E-04 | Sphinganine 1-phosphate |
| 8.91_377.1340m/z | Stunted | 3.02E-05 | 9.59E-04 | Galactinol dihydrate |
| 10.54_613.5250m/z | Stunted | 3.02E-05 | 9.59E-04 | Helianyl octanoate |
| 6.24_449.1816m/z | Normal | 3.02E-05 | 9.59E-04 | Geranylgeranyl-PP |
| 16.46_540.5336m/z | Normal | 3.51E-05 | 1.07E-03 | Cer(d18:0/16:0) |
| 9.19_283.2424m/z | Stunted | 3.51E-05 | 1.07E-03 | Cardanolmonoene |
| 9.36_400.1753n | Stunted | 3.51E-05 | 1.07E-03 | Corchoionoside B |
| 7.61_335.0498m/z | Normal | 4.07E-05 | 1.19E-03 | Aflatoxin B1 |
| 14.40_347.1092m/z | Normal | 4.07E-05 | 1.19E-03 | Muscomin |
| 9.92_355.2613m/z | Stunted | 4.07E-05 | 1.19E-03 | 2-(10-Heptadecenyl)-6-hydroxybenzoic acid |
| 7.61_160.0345m/z | Normal | 4.71E-05 | 1.32E-03 | Alloxan |
| 10.27_331.2636m/z | Stunted | 4.71E-05 | 1.32E-03 | 3,4-Dimethyl-5-pentyl-2-furanundecanoic acid |
| 9.43_388.2078m/z | Stunted | 4.71E-05 | 1.32E-03 | Levomethadyl Acetate |
| 17.92_427.2432m/z | Normal | 5.43E-05 | 1.47E-03 | Lovastatin |
| 13.29_527.4479m/z | Normal | 5.43E-05 | 1.47E-03 | Diepomuricanin A |
| 9.21_327.1259m/z | Stunted | 5.43E-05 | 1.47E-03 | Gibberellin A92 |
| 9.36_585.4928m/z | Stunted | 5.43E-05 | 1.47E-03 | Erythrinasinate A |
| 9.19_657.3582m/z | Stunted | 5.43E-05 | 1.47E-03 | Madlongiside C |
| 7.60_307.1035m/z | Normal | 6.26E-05 | 1.64E-03 | Starch acetate |
| 14.56_473.3962m/z | Normal | 6.26E-05 | 1.64E-03 | (3beta,4alpha,5alpha,14alpha,24xi)-4,14,24-Trimethylcholest-9(11)-en-3-ol |
| 7.82_436.2812m/z | Normal | 6.26E-05 | 1.64E-03 | L-Palmitoylcarnitine |
| 1.18_243.0615m/z | Stunted | 6.26E-05 | 1.64E-03 | tulobuterol |
| 7.61_247.1231m/z | Normal | 7.20E-05 | 1.85E-03 | Ergothioneine |
| 8.89_382.1686n | Stunted | 7.20E-05 | 1.85E-03 | Eletriptan |
| 6.25_460.2106m/z | Normal | 7.20E-05 | 1.85E-03 | Leukotriene E4 |
| 9.65_408.1130n | Stunted | 8.27E-05 | 2.01E-03 | 4-Methoxybenzenepropanol 1-(2-sulfoglucoside) |
| 2.78_146.9626m/z | Normal | 8.27E-05 | 2.01E-03 | Glycerol alpha-monochlorohydrin |
| 8.91_377.1479m/z | Stunted | 8.27E-05 | 2.01E-03 | 1-(3-Methylbutanoyl)-6-apiosylglucose |
| 5.71_447.1260m/z | Normal | 8.27E-05 | 2.01E-03 | Melledonal B |
| 7.83_464.3044m/z | Normal | 8.27E-05 | 2.01E-03 | 3a,7b,12a-Trihydroxyoxocholanyl-Glycine |
| 9.43_371.2190m/z | Stunted | 8.27E-05 | 2.01E-03 | Tussilagone |
| 9.64_415.2034m/z | Stunted | 8.27E-05 | 2.01E-03 | Xanthoplanine |
| 7.60_145.0379m/z | Normal | 9.49E-05 | 2.16E-03 | 2-Acetylpyrazine |
| 5.14_306.2543m/z | Normal | 9.49E-05 | 2.16E-03 | Levobupivacaine |
| 14.09_284.9499m/z | Normal | 9.49E-05 | 2.16E-03 | Zinc sulfate heptahydrate |
| 10.66_452.3451n | Stunted | 9.49E-05 | 2.16E-03 | 5b-Cholestane-3a,7a,12a,23S,25-pentol |
| 8.77_257.2270m/z | Stunted | 9.49E-05 | 2.16E-03 | 5a-Androstan-3b-ol |
| 1.50_188.0563m/z | Stunted | 9.49E-05 | 2.16E-03 | Dihydrolipoamide |
| 9.39_354.1504n | Stunted | 1.09E-04 | 2.34E-03 | 2,3-Dihydro-7-methoxy-2-(3-methoxy-4,5-methylenedioxyphenyl)-3-  methyl-5-(1-propenyl)benzofuran |
| 3.07_506.0910m/z | Normal | 1.09E-04 | 2.34E-03 | Cefotiam |
| 2.77_610.2773m/z | Normal | 1.09E-04 | 2.34E-03 | Mauritine A |
| 3.07_159.0915m/z | Normal | 1.09E-04 | 2.34E-03 | Nicotine-1'-N-oxide |
| 1.21_199.0274m/z | Stunted | 1.09E-04 | 2.34E-03 | ricinine |
| 8.32_279.1852m/z | Normal | 1.24E-04 | 2.62E-03 | Triprolidine |
| 9.38_562.3840n | Stunted | 1.24E-04 | 2.62E-03 | Cholesterol glucuronide |
| 2.78_382.0985m/z | Stunted | 1.24E-04 | 2.62E-03 | Succinyladenosine |
| 5.78_297.1116m/z | Stunted | 1.42E-04 | 2.85E-03 | Aspartyl-Tyrosine |
| 17.09_612.5926m/z | Normal | 1.42E-04 | 2.85E-03 | Ethylene glycol distearate |
| 10.55_667.4332m/z | Stunted | 1.42E-04 | 2.85E-03 | Glycerol 1-(9Z-octadecenoate) 2-tetradecanoate 3-phosphate |
| 5.69_448.1287m/z | Normal | 1.42E-04 | 2.85E-03 | Pemetrexed |
| 3.07_287.0352m/z | Normal | 1.42E-04 | 2.85E-03 | 2-O-(Z-p-Hydroxycinnamoyl)-(x)-glyceric acid |
| 4.45_171.1017m/z | Stunted | 1.62E-04 | 3.16E-03 | cis-3-Hexenyl lactate |
| 9.96_415.1639m/z | Stunted | 1.62E-04 | 3.16E-03 | Phenylethyl primeveroside |
| 8.91_387.1447m/z | Stunted | 1.62E-04 | 3.16E-03 | 8-Hydroxy-4'-methoxypinoresinol |
| 8.77_451.1431m/z | Stunted | 1.62E-04 | 3.16E-03 | Caryoptosidic acid |
| 3.06_186.0552m/z | Normal | 1.62E-04 | 3.16E-03 | Indolelactic acid |
| 9.93_455.1766m/z | Stunted | 1.84E-04 | 3.52E-03 | Atrovirinone |
| 0.93_673.2290m/z | Stunted | 1.84E-04 | 3.52E-03 | 6-Sialyl-N-acetyllactosamine |
| 6.55_413.1975m/z | Normal | 1.84E-04 | 3.52E-03 | Melleolide G |
| 9.64_384.2157n | Stunted | 2.09E-04 | 3.82E-03 | 20-Trihydroxy-leukotriene-B4 |
| 12.20_250.1391m/z | Normal | 2.09E-04 | 3.82E-03 | Fencamfamine |
| 7.88_269.2118m/z | Stunted | 2.09E-04 | 3.82E-03 | (S)-10,16-Dihydroxyhexadecanoic acid |
| 8.91_655.3652m/z | Stunted | 2.09E-04 | 3.82E-03 | 3-trans-p-Coumaroylrotundic acid |
| 6.14_227.0197m/z | Stunted | 2.37E-04 | 4.18E-03 | Bis(2-furanylmethyl) disulfide |
| 13.70_364.8985m/z | Normal | 2.37E-04 | 4.18E-03 | Di-2-propenyl heptasulfide |
| 12.55_312.3038m/z | Normal | 2.37E-04 | 4.18E-03 | Solacaproine |
| 8.77_301.2165m/z | Stunted | 2.37E-04 | 4.18E-03 | 11,12-EpETrE |
| 9.14_315.2327m/z | Stunted | 2.37E-04 | 4.18E-03 | 1-(3,5-Dihydroxyphenyl)-2-pentadecanone |
| 5.66_447.1337m/z | Normal | 2.68E-04 | 4.54E-03 | Agnuside |
| 5.51_249.1112m/z | Stunted | 2.68E-04 | 4.54E-03 | Kamahine C |
| 4.91_183.1382m/z | Stunted | 2.68E-04 | 4.54E-03 | 3-Hydroxynonyl acetate |
| 7.27_281.2474m/z | Stunted | 2.68E-04 | 4.54E-03 | (R)-3-Hydroxy-Octadecanoic acid |
| 9.65_399.1374n | Stunted | 3.04E-04 | 4.79E-03 | oxmetidine |
| 14.71_266.1503m/z | Normal | 3.04E-04 | 4.79E-03 | Valyl-Lysine |
| 5.67_448.1421m/z | Normal | 3.04E-04 | 4.79E-03 | Carphenazine |
| 9.21_443.1253m/z | Stunted | 3.04E-04 | 4.79E-03 | Mammea A/AC cyclo F |
| 10.46_357.2784m/z | Stunted | 3.04E-04 | 4.79E-03 | Isolithocholic acid |
| 7.79_194.0582m/z | Stunted | 3.04E-04 | 4.79E-03 | 4-Amino-2-methyl-1-naphthol |
| 5.68_448.1296m/z | Normal | 3.04E-04 | 4.79E-03 | Moricizine |
| 5.40_327.2180m/z | Stunted | 3.04E-04 | 4.79E-03 | Sorbitan laurate |
| 2.83_225.0880m/z | Stunted | 3.04E-04 | 4.79E-03 | (2S,4S)-Pinnatanine |
| 7.24_569.2781m/z | Normal | 3.04E-04 | 4.79E-03 | D-Urobilin |
| 8.91_729.3051m/z | Stunted | 3.04E-04 | 4.79E-03 | Scillipheosidin 3-[glucosyl-(1->2)-rhamnoside] |
| 7.60_226.0647n | Normal | 3.43E-04 | 5.23E-03 | 2-Methoxyxanthone |
| 14.16_527.2984m/z | Normal | 3.43E-04 | 5.23E-03 | Isodesmosine |
| 14.71_156.0072m/z | Normal | 3.43E-04 | 5.23E-03 | L-Threonine |
| 9.21_411.1106m/z | Stunted | 3.43E-04 | 5.23E-03 | 5,7-Dihydroxy-6-methoxyflavone 5-rhamnoside |
| 9.64_379.1613n | Stunted | 3.43E-04 | 5.23E-03 | 2-(Arabinosylamino)-3-(glucosylamino)propanenitrile |
| 2.66_296.0979m/z | Stunted | 3.43E-04 | 5.23E-03 | Nelarabine |
| 13.88_485.2909m/z | Normal | 3.87E-04 | 5.62E-03 | Desglucocoroloside |
| 9.29_683.3120m/z | Normal | 3.87E-04 | 5.62E-03 | Goshonoside F5 |
| 5.89_913.4840m/z | Normal | 3.87E-04 | 5.62E-03 | Hovenidulcioside B1 |
| 9.34_681.2810m/z | Normal | 3.87E-04 | 5.62E-03 | Myricanol 5-laminaribioside |
| 3.95_273.1244m/z | Stunted | 3.87E-04 | 5.62E-03 | Fusarochromanone |
| 0.96_128.0576n | Stunted | 3.87E-04 | 5.62E-03 | L-Cyclo(alanylglycyl) |
| 7.90_195.0894m/z | Normal | 4.36E-04 | 6.20E-03 | 3-[(2-Mercapto-1-methylpropyl)thio]-2-butanol |
| 3.71_131.0709m/z | Stunted | 4.36E-04 | 6.20E-03 | 2-Ethyl-2-Hydroxybutyric acid |
| 8.91_277.2168m/z | Stunted | 4.36E-04 | 6.20E-03 | Alpha-dimorphecolic |
| 8.92_657.3540m/z | Stunted | 4.36E-04 | 6.20E-03 | (S)-Nerolidol 3-O-[a-L-rhamnopyranosyl-(1->4)-a-L-rhamnopyranosyl-  (1->6)-b-D-glucopyranoside] |
| 0.94_256.9916m/z | Stunted | 4.36E-04 | 6.20E-03 | 1-Propenyl 1-(1-propenylthio)propyl disulfide |
| 7.82_536.2098m/z | Normal | 4.36E-04 | 6.20E-03 | Furcelleran |
| 5.97_395.2394m/z | Normal | 4.91E-04 | 6.58E-03 | Ascorbyl palmitate |
| 13.70_365.3427m/z | Normal | 4.91E-04 | 6.58E-03 | Cerebronic acid |
| 13.41_284.9571m/z | Normal | 4.91E-04 | 6.58E-03 | Methyl (Z)-5-(1-propynyl)-2-thienylacrylate |
| 9.28_682.2840m/z | Normal | 4.91E-04 | 6.58E-03 | S-(11-hydroxy-9-deoxy-delta12-PGD2)-glutathione |
| 9.78_1119.5055m/z | Stunted | 4.91E-04 | 6.58E-03 | AzII |
| 9.94_453.1689m/z | Stunted | 4.91E-04 | 6.58E-03 | 5-Formiminotetrahydrofolic acid |
| 6.10_459.0386m/z | Stunted | 4.91E-04 | 6.58E-03 | Cefuroxime |
| 9.29_653.2872m/z | Normal | 4.91E-04 | 6.58E-03 | Coagulin R 3-glucoside |
| 12.34_637.4356m/z | Normal | 4.91E-04 | 6.58E-03 | Ginsenoside F1 |
| 0.96_263.9799m/z | Stunted | 4.91E-04 | 6.58E-03 | Risedronate |
| 2.95_624.3024m/z | Normal | 4.91E-04 | 6.58E-03 | S-(9-deoxy-delta12-PGD2)-glutathione |
| 13.22_485.2701m/z | Normal | 5.51E-04 | 7.21E-03 | Asperagenin |
| 14.42_485.2807m/z | Normal | 5.51E-04 | 7.21E-03 | Neuromedin N (1-4) |
| 5.16_465.1451m/z | Normal | 5.51E-04 | 7.21E-03 | 7-Methyl-1,4,5-naphthalenetriol 4-[xylosyl-(1->6)-glucoside] |
| 5.34_449.1507m/z | Normal | 5.51E-04 | 7.21E-03 | Adapalene |
| 3.07_142.0655m/z | Normal | 5.51E-04 | 7.21E-03 | Tryptophanol |
| 14.17_415.1734m/z | Normal | 6.19E-04 | 7.78E-03 | Diltiazem |
| 14.40_290.1232n | Normal | 6.19E-04 | 7.78E-03 | N2-(3-Carboxy-2-hydroxy-1-oxopropyl)arginine |
| 13.15_377.1296m/z | Normal | 6.19E-04 | 7.78E-03 | Romucosine B |
| 4.67_380.1070m/z | Stunted | 6.19E-04 | 7.78E-03 | Rosiglitazone |
| 1.27_284.1258m/z | Normal | 6.19E-04 | 7.78E-03 | N5-Acetyl-N2-gamma-L-glutamyl-L-ornithine |
| 0.93_268.9909m/z | Stunted | 6.19E-04 | 7.78E-03 | Ajoene |
| 16.24_643.4240m/z | Stunted | 6.93E-04 | 8.61E-03 | Soyasapogenol B 24-O-b-D-glucoside |
| 9.31_682.2993m/z | Normal | 6.93E-04 | 8.61E-03 | Chitin |
| 8.77_203.1806m/z | Stunted | 6.93E-04 | 8.61E-03 | Sesquisabinene hydrate |
| 7.34_256.2397n | Normal | 7.76E-04 | 9.30E-03 | 2-Octanone |
| 12.68_332.8968m/z | Normal | 7.76E-04 | 9.30E-03 | Clofenotane |
| 2.29_158.9753m/z | Stunted | 7.76E-04 | 9.30E-03 | Allitridin |
| 9.30_681.2913m/z | Normal | 7.76E-04 | 9.30E-03 | Coproporphyrinogen III |
| 0.78_895.3418m/z | Stunted | 7.76E-04 | 9.30E-03 | Olitoriusin |
| 9.38_611.3693m/z | Stunted | 7.76E-04 | 9.30E-03 | Tocophersolan |
| 7.60_281.1287m/z | Normal | 8.68E-04 | 9.95E-03 | Asparaginyl-Methionine |
| 10.00_268.1377m/z | Normal | 8.68E-04 | 9.95E-03 | Voglibose |
| 9.22_655.4306m/z | Normal | 8.68E-04 | 9.95E-03 | Mosinone A |
| 9.23_311.1723m/z | Stunted | 8.68E-04 | 9.95E-03 | N-Undecylbenzenesulfonic acid |
| 3.81_811.4545m/z | Stunted | 8.68E-04 | 9.95E-03 | (3b,16a,20R)-3,16,20,22,25-Pentahydroxy-5-cucurbiten-11-one 3-[glucosyl-(1->6)-glucoside] |
| 9.66_434.1261m/z | Stunted | 8.68E-04 | 9.95E-03 | (卤)-alpha-Narcotine |
| 2.91_194.0442m/z | Stunted | 8.68E-04 | 9.95E-03 | Droxidopa |
| 5.31_465.1637m/z | Normal | 8.68E-04 | 9.95E-03 | Halobetasol Propionate |
| 2.84_317.0912m/z | Stunted | 8.68E-04 | 9.95E-03 | Chlorpromazine |
| 8.77_483.1283m/z | Stunted | 8.68E-04 | 9.95E-03 | N-[[3-(b-D-Glucopyranosyloxy)-2,3-dihydro-2-oxo-1H-indol-3-yl]acetyl]aspartic acid |
| 9.65_276.1613n | Normal | 9.69E-04 | 1.06E-02 | 4-Hydroxycinnamoylagmatine |
| 14.41_371.1594m/z | Normal | 9.69E-04 | 1.06E-02 | Thioridazine |
| 4.09_188.1046n | Stunted | 9.69E-04 | 1.06E-02 | (+/-)-Ethyl 3-acetoxy-2-methylbutyrate |
| 4.09_125.0964m/z | Stunted | 9.69E-04 | 1.06E-02 | 2-Ethylhexanoic acid |
| 3.07_117.0570n | Normal | 9.69E-04 | 1.06E-02 | Benzeneacetonitrile |
| 6.07_456.0486m/z | Stunted | 9.69E-04 | 1.06E-02 | Cyanidin 7-arabinoside |
| 5.29_155.8661m/z | Normal | 9.69E-04 | 1.06E-02 | Permanganate |
| 0.97_207.0505m/z | Stunted | 9.69E-04 | 1.06E-02 | 5-Acetylamino-6-formylamino-3-methyluracil |
| 8.61_580.2367m/z | Normal | 9.69E-04 | 1.06E-02 | N-[(3a,5b,7a,12a)-3,7-dihydroxy-24-oxo-12-(sulfooxy)cholan-24-yl]-Glycine |
| 2.72_245.1082m/z | Stunted | 9.69E-04 | 1.06E-02 | 2,3,5-Trimethyl-6-[4-(methylthio)butyl]pyrazine |
| 7.61_171.1032m/z | Normal | 1.08E-03 | 1.13E-02 | 1,4-Ipomeadiol |
| 7.82_414.2001n | Normal | 1.08E-03 | 1.13E-02 | Eplerenone |
| 12.06_297.2783m/z | Normal | 1.08E-03 | 1.13E-02 | 1,2,4-Nonadecanetriol |
| 8.62_480.3085m/z | Normal | 1.08E-03 | 1.13E-02 | 12-Hydroxy-12-octadecanoylcarnitine |
| 2.74_144.0450m/z | Normal | 1.08E-03 | 1.13E-02 | 3-Methyldioxyindole |
| 3.86_857.4828m/z | Stunted | 1.08E-03 | 1.13E-02 | Glabrin C |
| 7.62_452.2798m/z | Normal | 1.08E-03 | 1.13E-02 | Sambutoxin |
| 1.90_360.1005m/z | Stunted | 1.08E-03 | 1.13E-02 | Bezafibrate |
| 9.38_626.3460m/z | Stunted | 1.08E-03 | 1.13E-02 | Janthitrem F |
| 1.03_254.9803m/z | Stunted | 1.08E-03 | 1.13E-02 | Riluzole |
| 14.40_340.0973m/z | Normal | 1.20E-03 | 1.21E-02 | L-gamma-Glutamyl-S-allylthio-L-cysteine |
| 14.41_659.2928m/z | Normal | 1.20E-03 | 1.21E-02 | Paroxetine |
| 12.57_311.2940m/z | Normal | 1.20E-03 | 1.21E-02 | 5-Hydroxy-7-eicosanone |
| 7.64_557.3297m/z | Normal | 1.20E-03 | 1.21E-02 | Cyclopassifloic acid B |
| 9.19_659.3671m/z | Stunted | 1.20E-03 | 1.21E-02 | Gingerglycolipid B |
| 7.85_543.3378m/z | Normal | 1.20E-03 | 1.21E-02 | Hovenidulcigenin A |
| 9.43_442.1422n | Stunted | 1.20E-03 | 1.21E-02 | Methacycline |
| 2.53_373.0983m/z | Stunted | 1.20E-03 | 1.21E-02 | Triflupromazine |
| 14.41_777.4223m/z | Normal | 1.34E-03 | 1.31E-02 | Lyciumoside IV |
| 16.23_321.2010m/z | Stunted | 1.34E-03 | 1.31E-02 | Pimelylcarnitine |
| 9.38_177.9028m/z | Stunted | 1.34E-03 | 1.31E-02 | Cesium |
| 4.75_215.1287m/z | Stunted | 1.34E-03 | 1.31E-02 | Undecanedioic acid |
| 2.65_149.0457m/z | Stunted | 1.34E-03 | 1.31E-02 | D-Xylose |
| 5.11_724.1826m/z | Stunted | 1.34E-03 | 1.31E-02 | Cyanidin 3-sambubioside 5-glucoside |
| 2.69_287.0831m/z | Stunted | 1.34E-03 | 1.31E-02 | Fluconazole |
| 8.91_421.1001m/z | Stunted | 1.34E-03 | 1.31E-02 | Furaneol 4-(6-malonylglucoside) |
| 5.01_218.2001m/z | Normal | 1.49E-03 | 1.41E-02 | 2-Hexenoylcholine |
| 5.70_415.2934m/z | Normal | 1.49E-03 | 1.41E-02 | Phytosphingosine-1-P |
| 3.22_251.0374m/z | Normal | 1.49E-03 | 1.41E-02 | Taurine |
| 10.02_277.1251m/z | Normal | 1.49E-03 | 1.41E-02 | Triethyl citrate |
| 6.07_456.2418m/z | Stunted | 1.49E-03 | 1.41E-02 | 10,11-Dihydro-12R-hydroxy-leukotriene E4 |
| 0.96_153.0301m/z | Stunted | 1.49E-03 | 1.41E-02 | L-alpha-Amino-5-oxo-2(5H)-isoxazolepropanoic acid |
| 8.94_396.1201m/z | Stunted | 1.49E-03 | 1.41E-02 | Adinazolam |
| 2.09_345.1312m/z | Normal | 1.49E-03 | 1.41E-02 | Gibberellin A72 |
| 9.38_601.3268m/z | Stunted | 1.49E-03 | 1.41E-02 | Panaxydol linoleate |
| 0.72_402.9177m/z | Normal | 1.49E-03 | 1.41E-02 | Fructose 1,6-bisphosphate |
| 7.14_295.1079m/z | Normal | 1.65E-03 | 1.53E-02 | Rosoxacin |
| 6.19_431.1917m/z | Normal | 1.65E-03 | 1.53E-02 | sofalcone |
| 4.21_225.0710m/z | Stunted | 1.65E-03 | 1.53E-02 | Biotin |
| 3.77_768.4436m/z | Stunted | 1.65E-03 | 1.53E-02 | Clarithromycin |
| 3.69_665.3565m/z | Stunted | 1.83E-03 | 1.67E-02 | Coroloside |
| 9.65_363.1888m/z | Stunted | 1.83E-03 | 1.67E-02 | 16-Methylheptadecanoic acid |
| 14.47_376.3097m/z | Stunted | 2.03E-03 | 1.81E-02 | 13,14-Dihydro PGF-1a |
| 8.87_308.1895n | Normal | 2.03E-03 | 1.81E-02 | Indecainide |
| 0.71_164.9335m/z | Normal | 2.03E-03 | 1.81E-02 | Methyl 2-propenyl tetrasulfide |
| 5.88_913.4607m/z | Normal | 2.03E-03 | 1.81E-02 | Dirithromycin |
| 8.91_612.3664n | Stunted | 2.03E-03 | 1.81E-02 | (24E)-3beta,15alpha,22S-Triacetoxylanosta-7,9(11),24-trien-26-oic acid |
| 9.63_414.1055m/z | Stunted | 2.03E-03 | 1.81E-02 | imidaprilat |
| 16.24_347.1634m/z | Stunted | 2.24E-03 | 1.92E-02 | N-trans-Feruloyloctopamine |
| 7.60_251.1330m/z | Normal | 2.24E-03 | 1.92E-02 | Threoninyl-Asparagine |
| 8.62_481.3030m/z | Normal | 2.24E-03 | 1.92E-02 | Campestanol |
| 9.25_311.1639m/z | Stunted | 2.24E-03 | 1.92E-02 | Carnosol |
| 7.53_295.2283m/z | Stunted | 2.24E-03 | 1.92E-02 | (9xi,10xi,12xi)-9,10-Dihydroxy-12-octadecenoic acid |
| 4.22_214.1424m/z | Stunted | 2.24E-03 | 1.92E-02 | N-Nonanoylglycine |
| 3.61_456.1995m/z | Stunted | 2.24E-03 | 1.92E-02 | Valsartan |
| 5.61_448.2981m/z | Normal | 2.24E-03 | 1.92E-02 | tiropramide |
| 6.16_456.0445m/z | Stunted | 2.24E-03 | 1.92E-02 | Cloxacillin |
| 6.46_478.1638m/z | Normal | 2.24E-03 | 1.92E-02 | Minocycline |
| 5.13_768.2142m/z | Stunted | 2.24E-03 | 1.92E-02 | Peonidin 3-diglucoside 5-glucoside |
| 8.92_417.1229m/z | Stunted | 2.24E-03 | 1.92E-02 | Phenethyl 6-galloylglucoside |
| 5.99_237.1495m/z | Stunted | 2.48E-03 | 2.06E-02 | 5-Nonyltetrahydro-2-oxo-3-furancarboxylic acid |
| 9.19_839.5521m/z | Stunted | 2.48E-03 | 2.06E-02 | all-trans-decaprenyl diphosphate |
| 9.43_282.2579n | Stunted | 2.48E-03 | 2.06E-02 | Elaidic acid |
| 6.59_431.3116m/z | Stunted | 2.48E-03 | 2.06E-02 | Coprocholic acid |
| 1.20_167.0204m/z | Stunted | 2.48E-03 | 2.06E-02 | Uric acid |
| 10.93_333.2801m/z | Stunted | 2.48E-03 | 2.06E-02 | (all-Z)-7,10,13-Docosatrienoic acid |
| 3.64_783.4204m/z | Stunted | 2.48E-03 | 2.06E-02 | 1alpha,3beta,22R-Trihydroxyergosta-5,24E-dien-26-oic acid  3-O-b-D-glucoside 26-O-b-D-glucosyl ester |
| 2.91_100.0036m/z | Stunted | 2.48E-03 | 2.06E-02 | Aminomalonic acid |
| 2.83_331.1054m/z | Stunted | 2.48E-03 | 2.06E-02 | Moschamindole |
| 9.39_628.3432m/z | Stunted | 2.48E-03 | 2.06E-02 | Nummularine A |
| 3.59_706.1587m/z | Stunted | 2.48E-03 | 2.06E-02 | p-Coumaroyl vitisin A |
| 9.19_329.1262m/z | Stunted | 2.74E-03 | 2.21E-02 | (卤)-threo-1-(p-Hydroxyphenyl)propylene glycol 4'-glucoside |
| 4.76_448.3039m/z | Normal | 2.74E-03 | 2.21E-02 | Chenodeoxyglycocholic acid |
| 9.64_664.3917n | Stunted | 2.74E-03 | 2.21E-02 | 3-O-trans-Feruloyleuscaphic acid |
| 3.86_206.0827m/z | Stunted | 2.74E-03 | 2.21E-02 | Cytokinin B |
| 1.40_279.0611m/z | Stunted | 2.74E-03 | 2.21E-02 | Nicotinamide ascorbate |
| 3.83_1003.5521m/z | Stunted | 2.74E-03 | 2.21E-02 | Phaseoluside A |
| 0.96_316.1140m/z | Normal | 2.74E-03 | 2.21E-02 | Tryptophyl-Methionine |
| 9.63_407.1039n | Stunted | 3.02E-03 | 2.40E-02 | Erysothiopine |
| 3.51_669.3036m/z | Stunted | 3.02E-03 | 2.40E-02 | (3b,20R,22R)-3,20,27-Trihydroxy-1-oxowitha-5,24-dienolide 3-glucoside |
| 3.59_222.0648n | Normal | 3.02E-03 | 2.40E-02 | Allocystathionine |
| 0.89_325.0253m/z | Stunted | 3.02E-03 | 2.40E-02 | Molybdopterin precursor Z |
| 7.34_670.2806m/z | Normal | 3.02E-03 | 2.40E-02 | Glycylserylprolylmethionylphenylalanylvalinamide |
| 14.41_243.0667m/z | Normal | 3.32E-03 | 2.56E-02 | 2,4,5,6-Phenanthrenetetrol |
| 17.08_580.4325m/z | Normal | 3.32E-03 | 2.56E-02 | Cholesteryl ferulate |
| 5.82_252.0076m/z | Stunted | 3.32E-03 | 2.56E-02 | Clonidine |
| 6.15_195.1381m/z | Normal | 3.32E-03 | 2.56E-02 | 3-Oxododecanoic acid |
| 4.31_240.0873m/z | Stunted | 3.32E-03 | 2.56E-02 | Osmaronin |
| 3.76_915.5000m/z | Stunted | 3.32E-03 | 2.56E-02 | (3b,5b,22a,25R)-Furostane-22-methoxy-3,26-diol 3-[glucosyl-(1->2)-glucoside] 26-glucoside |
| 0.99_119.0345m/z | Stunted | 3.32E-03 | 2.56E-02 | (S)-3,4-Dihydroxybutyric acid |
| 7.96_279.2307m/z | Normal | 3.32E-03 | 2.56E-02 | 11-Oxooctadecanoic acid |
| 9.46_371.2112m/z | Stunted | 3.32E-03 | 2.56E-02 | 7,8-Dihydro-3b,6a-dihydroxy-alpha-ionol 9-glucoside |
| 3.42_404.0397m/z | Stunted | 3.32E-03 | 2.56E-02 | Cephapirin |
| 6.07_586.0020m/z | Stunted | 3.32E-03 | 2.56E-02 | Formamidopyrimidine nucleoside triphosphate |
| 9.16_329.2338m/z | Stunted | 3.32E-03 | 2.56E-02 | Oxyphenonium |
| 5.55_329.1402m/z | Stunted | 3.66E-03 | 2.77E-02 | Acutilobin |
| 3.89_899.5037m/z | Stunted | 3.66E-03 | 2.77E-02 | (3b,21b)-12-Oleanene-3,21,28-triol 28-[arabinosyl-(1->3)-arabinosyl-(1->3)-arabinoside] |
| 7.58_543.3414m/z | Normal | 3.66E-03 | 2.77E-02 | 2'-Apo-beta-carotenal |
| 9.78_427.1711m/z | Stunted | 3.66E-03 | 2.77E-02 | 5'-O-Methylmelledonal |
| 5.04_546.2095m/z | Normal | 3.66E-03 | 2.77E-02 | D-Glucosaminide |
| 7.18_292.0930m/z | Normal | 4.02E-03 | 2.99E-02 | 5-Hydroxythalidomide |
| 7.56_481.2338m/z | Normal | 4.02E-03 | 2.99E-02 | 1,25-Dihydroxyvitamin D3-26,23-lactone |
| 9.29_311.1742m/z | Stunted | 4.02E-03 | 2.99E-02 | Praziquantel |
| 6.07_473.0555m/z | Normal | 4.02E-03 | 2.99E-02 | Torvanol A |
| 14.21_597.4882m/z | Normal | 4.02E-03 | 2.99E-02 | Cohibin C |
| 0.98_187.1084m/z | Stunted | 4.02E-03 | 2.99E-02 | Alanyl-Valine |
| 1.90_281.9743m/z | Stunted | 4.02E-03 | 2.99E-02 | nitrofen |
| 14.41_187.0035m/z | Normal | 4.41E-03 | 3.20E-02 | 8-Chloroxanthine |
| 14.12_655.3207m/z | Normal | 4.41E-03 | 3.20E-02 | Argenteane |
| 9.22_350.1701m/z | Normal | 4.41E-03 | 3.20E-02 | Zanamivir |
| 11.54_215.0015m/z | Stunted | 4.41E-03 | 3.20E-02 | 4-[2,2'-Bithiophen-5-yl]-3-butyn-1-ol |
| 9.20_227.2011m/z | Stunted | 4.41E-03 | 3.20E-02 | 8,8-Diethoxy-2,6-dimethyl-2-octanol |
| 10.40_423.1694m/z | Stunted | 4.41E-03 | 3.20E-02 | Lusitanicoside |
| 4.43_827.4107m/z | Stunted | 4.41E-03 | 3.20E-02 | Digitoxigenin 3-[glucosyl-(1->6)-glucosyl-(1->4)-2,6-dideoxyribohexoside] |
| 1.90_254.9516m/z | Stunted | 4.41E-03 | 3.20E-02 | Ribose-1-arsenate |
| 3.14_463.1842m/z | Stunted | 4.41E-03 | 3.20E-02 | Eremopetasitenin B2 |
| 3.39_574.0829m/z | Stunted | 4.41E-03 | 3.20E-02 | Mezlocillin |
| 11.90_325.1828m/z | Stunted | 4.84E-03 | 3.45E-02 | (E,E)-Boviquinone 3 |
| 7.67_541.3231m/z | Normal | 4.84E-03 | 3.45E-02 | Cucurbitacin C |
| 9.71_491.3221m/z | Normal | 4.84E-03 | 3.45E-02 | Ganodermic acid TQ |
| 11.15_359.2925m/z | Stunted | 4.84E-03 | 3.45E-02 | Tetracosatetraenoic acid (24:4n-6) |
| 4.97_449.3083m/z | Normal | 4.84E-03 | 3.45E-02 | Uralenolide |
| 12.19_551.3333m/z | Normal | 5.30E-03 | 3.72E-02 | (22S)-Acetoxy-3alpha,15alpha-dihydroxylanosta-7,9(11),24-trien-26-oic acid |
| 14.41_351.1638m/z | Normal | 5.30E-03 | 3.72E-02 | Eremopetasin sulfoxide |
| 13.01_167.0946m/z | Stunted | 5.30E-03 | 3.72E-02 | Sevelamer |
| 13.81_339.3254m/z | Normal | 5.30E-03 | 3.72E-02 | 4-Hydroxy-6-docosanone |
| 10.58_449.1794m/z | Stunted | 5.30E-03 | 3.72E-02 | Taraxacolide 1-O-b-D-glucopyranoside |
| 9.38_609.3741m/z | Stunted | 5.30E-03 | 3.72E-02 | 22-Angeloyltheasapogenol A |
| 0.90_347.0502m/z | Stunted | 5.30E-03 | 3.72E-02 | Demethoxykanugin |
| 14.84_355.0621m/z | Stunted | 5.81E-03 | 4.00E-02 | 2-O-p-Coumaroylhydroxycitric acid |
| 5.41_288.2067m/z | Stunted | 5.81E-03 | 4.00E-02 | Doxylamine |
| 8.91_725.3583m/z | Stunted | 5.81E-03 | 4.00E-02 | Atazanavir |
| 9.21_494.1375m/z | Stunted | 5.81E-03 | 4.00E-02 | Pteroyl-D-glutamic acid |
| 3.59_201.9928m/z | Normal | 5.81E-03 | 4.00E-02 | 2,6-Pyridinedicarboxylic acid |
| 9.21_557.3455m/z | Stunted | 5.81E-03 | 4.00E-02 | Collettiside I |
| 6.60_634.2376m/z | Normal | 5.81E-03 | 4.00E-02 | ferrioxamine |
| 0.91_155.0009m/z | Stunted | 5.81E-03 | 4.00E-02 | 2,5-Dimethyl-3-(methyldithio)furan |
| 3.64_714.2087m/z | Stunted | 5.81E-03 | 4.00E-02 | Malvidin 3-laminaribioside |
| 2.83_152.9796m/z | Stunted | 6.35E-03 | 4.28E-02 | 2-Methyl-3-thiophenethiol |
| 2.76_161.0588m/z | Stunted | 6.35E-03 | 4.28E-02 | 6-Methylcoumarin |
| 6.62_504.2383m/z | Normal | 6.35E-03 | 4.28E-02 | Glucosylgalactosyl hydroxylysine |
| 9.43_205.1966m/z | Stunted | 6.35E-03 | 4.28E-02 | 3-Pentadecenal |
| 4.10_541.2603m/z | Stunted | 6.35E-03 | 4.28E-02 | Cortolone-3-glucuronide |
| 3.58_314.0403n | Normal | 6.35E-03 | 4.28E-02 | Laccaic acid D |
| 1.23_256.0925m/z | Stunted | 6.35E-03 | 4.28E-02 | Norophthalmic acid |
| 3.47_503.2678m/z | Stunted | 6.35E-03 | 4.28E-02 | Physangulide |
| 9.44_443.1440m/z | Stunted | 6.35E-03 | 4.28E-02 | Tetracycline |
| 1.22_204.1121m/z | Stunted | 6.94E-03 | 4.57E-02 | Tryptophanamide |
| 13.14_325.3103m/z | Normal | 6.94E-03 | 4.57E-02 | 6-Hydroxy-4-heneicosanone |
| 9.22_364.1072n | Stunted | 6.94E-03 | 4.57E-02 | Bumetanide |
| 8.91_451.0729m/z | Stunted | 6.94E-03 | 4.57E-02 | (Z)-Resveratrol 3-(2''-sulfoglucoside) |
| 2.58_151.0260m/z | Stunted | 6.94E-03 | 4.57E-02 | 6,8-Dihydroxypurine |
| 2.95_375.1289m/z | Stunted | 6.94E-03 | 4.57E-02 | 1-(3-Methyl-2-butenoyl)-6-apiosylglucose |
| 3.60_581.3010m/z | Stunted | 6.94E-03 | 4.57E-02 | Corchorosol A |
| 3.53_727.3428m/z | Stunted | 6.94E-03 | 4.57E-02 | Hordatine A glucoside |
| 2.66_243.1341m/z | Normal | 6.94E-03 | 4.57E-02 | Hydroxyprolyl-Isoleucine |
| 1.90_273.0248m/z | Stunted | 6.94E-03 | 4.57E-02 | Laccarin |
| 14.79_555.2899m/z | Stunted | 7.57E-03 | 4.87E-02 | Ganoderic acid I |
| 12.26_547.2732m/z | Normal | 7.57E-03 | 4.87E-02 | Morellinol |
| 3.13_279.1350m/z | Stunted | 7.57E-03 | 4.87E-02 | Phenylalanyl-Hydroxyproline |
| 12.87_138.0145m/z | Normal | 7.57E-03 | 4.87E-02 | (卤)-2-Methylthiazolidine |
| 3.73_173.0813m/z | Stunted | 7.57E-03 | 4.87E-02 | 1,9-Nonanedithiol |
| 8.31_719.4455m/z | Normal | 7.57E-03 | 4.87E-02 | (3b,22a)-12-Oleanene-3,22,24,29-tetrol 3-[arabinosyl-(1->3)-arabinoside] |
| 3.58_343.0663m/z | Normal | 7.57E-03 | 4.87E-02 | Thiamine monophosphate |
| 8.91_327.2223n | Stunted | 7.57E-03 | 4.87E-02 | Butorphanol |

**Supplementary Table 6. Differential metabolite features identified in both discovery and validation cohort**

| **Retention time (RT)–m/z** | **Group** | **FDR** | **Putative compound** |
| --- | --- | --- | --- |
| 14.86_332.8978m/z | Normal | 7.23E-04 | Clofenotane |
| 9.18_555.9811m/z | Normal | 9.59E-04 | Cefotetan |
| 14.72_485.2826m/z | Normal | 2.34E-03 | Neuromedin N (1-4) |
| 12.06_297.2783m/z | Normal | 1.13E-02 | 1,2,4-Nonadecanetriol |
| 3.22_251.0374m/z | Normal | 1.41E-02 | Taurine |
| 0.71_164.9335m/z | Normal | 1.81E-02 | Methyl 2-propenyl tetrasulfide |
| 7.56_152.9952m/z | Normal | 4.28E-02 | Glycerol 3-phosphate |
| 11.12_210.9992m/z | Stunted | 6.06E-04 | D-Xylulose 1-phosphate |
| 5.48_440.0524m/z | Stunted | 7.88E-04 | De-O-methylsimmondsin |
| 7.99_269.2121m/z | Stunted | 1.19E-03 | (S)-10,16-Dihydroxyhexadecanoic acid |
| 1.50_188.0563m/z | Stunted | 2.16E-03 | Dihydrolipoamide |
| 5.51_249.1112m/z | Stunted | 4.54E-03 | Kamahine C |
| 0.96_128.0576n | Stunted | 5.62E-03 | L-Cyclo(alanylglycyl) |
| 2.29_158.9753m/z | Stunted | 9.30E-03 | Allitridin |
| 1.40_128.0347m/z | Stunted | 1.81E-02 | L-Glutamic acid |
| 14.84_355.0621m/z | Stunted | 4.00E-02 | 2-O-p-Coumaroylhydroxycitric acid |
